# *Chloroxylon swietenia* (Roxb.) DC induces cell death and apoptosis by down‐regulating the NF‐κB pathway in MCF‐7 breast cancer cells: In vitro and in vivo investigations

**DOI:** 10.1002/cnr2.1600

**Published:** 2022-03-11

**Authors:** Sonali S. Kamble, Jasoda Choudhari, Ramakrishna Nimma, Totakura Venkata Santosh Kumar, Kapil K. Patil, Shrikant V. Hese, Bhaskar S. Dawane, Rajesh N. Gacche

**Affiliations:** ^1^ Department of Biochemistry Gramin Science (Vocational) College Nanded India; ^2^ Department of Biotechnology Savitribai Phule Pune University Pune India; ^3^ National Centre for Cell Science Pune India; ^4^ Government Medical College & Hospital Aurangabad India; ^5^ DD Bhoyar College of Arts and Science Mouda Nagpur India; ^6^ School of Chemical Sciences Swami Ramanand Teerth Marathwada University Nanded India

**Keywords:** apoptosis, breast cancer, *Chloroxylon swietenia*, cytotoxicity, MCF‐7, NF‐κB pathway

## Abstract

**Background:**

Natural products with targeted bioactivity have gained major attention in the field of cancer research owing to emerging anti‐cancer drug resistance and off target toxicities. *Chloroxylon swietenia* (Roxb.) DC is recognized as a folklore medicinal plant and has numerous therapeutic benefits in the folklore medicine system, however the anti‐cancer potential of this plant and its mechanism of action is poorly understood.

**Aims:**

The aim of the study was to investigate the anti‐breast cancer efficacy of *C. swietenia* leaves methanol extract (CSLME) against MCF‐7 hormone dependent human breast cancer cell line with possible mechanism of action.

**Methods and results:**

The anti‐breast cancer activity of CSLME against MCF‐7 cells was assessed by evaluating its efficacy toward cytotoxicity, cell migration, colony formation, DNA fragmentation, apoptosis, cytoskeleton, angiogenesis, cell cycle regulation, and animal toxicity. The preliminary screening of CSLME against MCF‐7 cells revealed the cytotoxicity (IC_50_ 20 μg/ml), inhibited cell migration, colony formation, and angiogenesis. It was observed that CSLME induces apoptosis by nuclear fragmentation and disruption of cytoskeleton by actin derangement. The results of Annexin V‐FITC assay and cell cycle analysis by flow cytometry clearly pointed out the sizable fraction of apoptotic cells, and arrested the cells at G2/M phase of cell cycle. The results of the immunoblotting experiments showed that CSLME activates intrinsic pathway of apoptosis with down regulation of anti‐apoptotic marker like Bcl2, up regulation of pro‐apoptotic markers like Bax & Bad, along with successful cleavage of Caspase‐9 and PARP‐1. Further, western blot analysis revealed the possible down regulation of NF‐κB pathway by CSLME, which may be responsible for anti‐cancer activity in MCF‐7 cells. In vivo animal model studies using NOD‐SCID mice demonstrated impressive anti‐tumor activity with significant reduction in tumor volume of MCF‐7 tumor xenograft. Of note, in‐vivo acute oral toxicity study as per Organization for Economic Cooperation and Development 423 revealed the nontoxic nature of CSLME.

**Conclusion:**

The in vitro and in vivo findings clearly outline the potential of CSLME as inhibitor of growth and proliferation of MCF‐7 cells. Mechanistically, CSLME seems to activate intrinsic pathway of apoptosis, arrest cell cycle, target actin cytoskeleton, inhibit growth, colony formation, migration, and angiogenesis, with down regulation of NF‐κB pathway leading to cell death.

## 1 INTRODUCTION

Breast cancer is the principal health issue among all gynecological cancers which afflicts massive population throughout the world.^1^ There are an estimated one million females diagnosed with invasive type of breast cancer every year worldwide and the disease is becoming the second most prominent reason for cancer deaths among women after lung cancer.^2^, ^3^ More precisely around 40 610 deaths of breast cancer among US women were estimated in 2017 while, in India the numbers of breast cancer cases were rising annually.^4^, ^5^ Although the breast cancer is specified as a single disease, it was classified into various histological subtypes. Based on the presence or absence of hormone receptors (HR) and human epidermal growth factor receptor 2 (HER2) breast cancer was further categorized among four different molecular subtypes such as Luminal A (HR+/HER2‐), Triple‐negative breast cancer (HR‐/HER2‐), HER2‐enriched (HR‐/HER2+), and Luminal B (HR+/HER2+).^4^ This differential expression pattern of hormones and growth factor is associated with the treatment response and prognosis of breast cancer patients.

Surgery, chemotherapy, and radiotherapy are the currently available treatment modalities for the management of breast cancer.^6^ Among these chemotherapy is the most widely used treatment strategy for breast cancer treatment; however, breast cancer is eminently resistant toward chemotherapy and till now the effective approach to cure the patients in an advanced stage is not available.^7^ Amid emerging drug resistance and developing breast cancer heterogeneity, the latest chemotherapy methods used in the treatment of hormone‐dependent breast cancers have drawbacks due to serious target and non‐target side effects. Therefore, the scientific quest is growing toward exploring novel, safe, and effective therapeutic agents for the management of breast cancer.

On the eve of emerging drug resistance and off target toxicities, natural resources have remained a ray of hope toward the drug discovery and therapeutic modality for a variety of human ailments including cancer. Nevertheless, over 33% of anticancer medications that are available are either natural products or their derivatives.^8^ The 2015 Nobel Prize appreciation to the natural product scientist has fueled the area of “natural product and drug discovery” research. Owing to the multi‐targeted and multi‐treatment potentials of natural resources/products, there is a renewed and evolving interest in the area of natural product research.^9^ The utilization of plant extracts and plant‐derived natural products as remedies existed thousands of years ago.^10^ Phytoconstituents have achieved significant recognition in the management of numerous human clinical complications owing to their specificity toward the molecular and cellular targets.^11^ Plants are always been a principal resource of extremely effective phytochemicals that furnish enormous potential to combat a number of diseases including cancer. Of note, plant‐derived natural products possess low toxicity and potent pharmacological actions, which perhaps are the benefits of exploring them as anticancer agents.^12^ Several plant species, comprising *Taxus baccata* (Taxol), *Podophyllum peltatum* (Podophyllotoxin), *Vinca rosea* (Vincristin & Vinblastin), and *Camptotecha accuminata* (Compothecin) are some of the milestone plant‐based FDA approved anti‐cancer drugs currently prescribed against a variety of cancers including breast. Moreover, a series of plant derived purified bioactive compounds are either under clinical trials or available as anticancer drugs.^13^ Therefore isolation, purification, and characterization of natural agents with anticancer properties are crucial for the invention of novel anticancer leads.

*Chloroxylon swietenia* (Roxb.) DC (*C. swietenia*) is a tree that belongs to the family of Rutaceae. This plant is native to India and Sri Lanka and is generally recognized as “Ceylon Satinwood or East Indian Satinwood.” *C. swietenia* is recognized as a folklore medicinal plant and has numerous medicinal benefits in the folklore medicine system.^14^ The stem bark is attributed to its efficiency to cure a common cold, cough, and ophthalmic infections.^15^ The bark is also used as an astringent while leaves are used for the treatment of inflammation‐associated disorders such as rheumatism. *C. swietenia* is also identified for potential activity against insects, pest, and beetles.^16^ The plant extract has been demonstrated to hold larvicidal, mosquito repellent, anti‐inflammatory, hepatoprotective, antimicrobial, anti‐diabetic, and antioxidant potential.^17^, ^18^ Taking into account the traditional importance of the plant and since the role of *C. swietenia* against breast cancer has not been reported yet, we inspired to undertake the current investigation of evaluating the anti‐breast cancer ability of *C. swietenia* leaves methanol extract (CSLME) against the MCF‐7 breast cancer cell line with a possible mode of action.

## 2 MATERIALS AND METHODS

### 2.1 Cells and cell culture

Breast cancer cell line (MCF‐7) was procured from the National Centre for Cell Sciences (NCCS), Pune, Maharashtra state, India. The cells were grown in Dulbecco's Modified Eagle Medium (DMEM), supplemented with 10% fetal bovine serum (FBS), 2 mM l‐glutamine, 100 mg/L penicillin, and 100 mg/L streptomycin. Cells were maintained in incubator at 37°C containing 5% CO_2_, 95% air along with 100% relative humidity. Cells were passaged weekly and the culture medium was changed twice a week. Authentication of the cell line was done by using Short Tandem Repeat analysis and Mycoplasma Testing.

### 2.2 Collection of the plant material, identification, and sample preparation

The *C. swietenia* leaves were collected from the nearby forest of “Kinwat city” of Nanded district (MS) India. RNG has verified the botanical authentication and identification of the *C. swietenia* plant and voucher specimen was deposited in the herbarium of the SRTM University (Botany Department), Nanded (MS), India. The fresh plant leaves were first washed followed by air‐drying in shade and then mechanically powdered. Powdered leaves were extracted in methanol solvents by operating Soxhlet's extraction equipment for 8 h at 60°C. Afterward, the solvent extract was evaporated in a rotary evaporator under vacuum. Then extract was stored at −20°C for further use.

### 2.3 Qualitative and quantitative screening of CSLME

CSLME was qualitatively tested by using the different qualitative measures as outlined earlier in standard experimental protocols to detect a range of phytochemicals such as tannins, terpenoids, saponins, and alkaloids.^19^ The total phenol content present in CSLME was estimated using Folin–Ciocalteu (FC) reagent.^20^ In summary, 500 μl (1 mg/ml) of CSLME solution was combined with 10% FC reagent (1.5 ml). A 3 ml of 7.5% Na_2_CO_3_ solution has been added after 5 min. The reaction blend has been incubated for 2 h at 30°C. The mixture absorption was estimated at 760 nm after 30 min of incubation. A standard curve of gallic acid was used to assess the phenol content and the phenol level was represented as gallic acid equivalents per gram of dry weight of the test extract (mg GAE/g DW). The amount of total flavonoid content in CSLME was calculated by the previously defined colorimetric aluminum chloride (AlCl_3_) method.^20^ One ml of CSLME (1 mg/ml), 1 M of potassium acetate (0.2 ml), 10% AlCl_3_ (0.2 ml), distilled water (3.8 ml), and 3 ml of methanol were combined. The absorption of the reaction mixture after 30 min of incubation was measured to be at 420 nm. A catechin standard curve was used to quantify the flavonoid content and this amount was calculated as catechin equivalents per gram of dry weight of test extract (mg CAE/g DW).

### 2.4 GC–MS analysis of CSLME

GC–MS analysis was performed to unravel the chemo‐profile of the CSLME which will also serve as a measure of quality control. The GC–MS analysis was conducted at Sophisticated Analytical Instrument Facility (SAIF), IIT Bombay, Mumbai, Maharashtra, India. The analysis was carried out using the Jeol spectrometer (Model: Accu TOF GCV). An electron ionization device with an ionization power of 70 eV was used for GC–MS study in “Electron Impact mode.” The carrier gas (helium 99.999%) with a consistent rate of flow of 1 ml/min was used, and 2 μl of injection volume is used (with 10:1 of split ratio). The injector temperature was maintained at 250°C, the temperature of ion source has been held to 220°C, the temperature of the oven has been set from 100°C (isothermic for 2 min), with a 10°C/min rise to 240°C, then 5°C/min to 280°C and an isothermal rise of 9 min at 280°C. At a scan‐interval of 0.4 s, mass spectra were reported. The solvent delay was nearly 0–4 min and the overall operating time of GC–MS was 35 min. The relative amount (%) of individual phytoconstituents was calculated by comparing its average peak area to the total areas. Phytochemicals were identified in their National Institute of Standard and Technology (NIST) library, by comparing the spectrum of unknown compounds with the spectrum of known compounds and the name, structure, and molecular weight of the compounds were probably confirmed.

### 2.5 MTT assay

In vitro MTT testing was done in accordance with the standard method mentioned earlier to determine CSLME's effect on breast cancer cells.^21^ Inoculation of the MCF‐7 cells at 1 × 10^5^ cells/ml density was carried out in 96 well culture plates. The cells were treated with different CSLME concentrations dissolved in 0.1% DMSO (Thermo Fisher Scientific, #D12345), and incubated for 48 h. The 20 μl MTT (2 mg/ml; ThermoFisher Scientific, #M6494), was applied to each well after the incubation time and the cells were incubated further at 37°C for 4 h. Further, formazan crystals were dissolved in isopropanol and the amount of formazan produced was estimated at 570 nm. The required concentration for inhibition of 50% cell viability has been calculated as IC_50_.

### 2.6 Effect of CSLME on cell migration: analysis using scratch and transmigration assay

The MCF‐7 cell migration was determined using the wound healing experiment as per the earlier described method.^22^ In brief, 2 × 10^5^ cells/well were seeded in 12 well cell culture plates and incubated with DMEM medium for 24 h. The sterile pipette tip of 10 μl was used to generate scratch on a cell monolayer. Detached cells have been separated by washing the cell monolayer with phosphate buffer saline (PBS). The CSLME (20 μg/ml) containing complete medium was added. The pictures were taken at 0 and 48 h with a digital camera on an inverted Nikon microscope. The migrated area was measured using NIS‐elements BR analysis Software.

The transwell migration experiment was performed by using the “Transwell Cell Culture Chamber” (Sigma‐Aldrich, #3464) as described elsewhere.^23^ In brief, the 5 × 10^5^ MCF‐7 cells were seeded in 60 mm cell culture plates and treated for 24 h using either DMSO (vehicle control: VC) or CSLME (20 μg/ml). VC and CSLME treated cells were collected by trypsinization and 2 × 10^5^ cells were added in the upper compartment containing pre‐hydrated polycarbonate membrane filter with a serum‐free media. The lower section was filled with DMEM medium and 10% FBS, which acts as a chemoattractant. The cells were allowed to migrate toward the lower chamber for 24 h and the non‐migrated cells that were present on the upper chamber were removed by extensive scraping. Further the migrated cells, on the contrary, are stained using 1% crystal violet stain. Images were captured under Nikon microscope, and the number of cells migrated was measured with Image J software.

### 2.7 CSLME effect on colony formation (clonogenic assay)

The assay was conducted in accordance with the procedure mentioned earlier.^24^ In the 6‐well plate, the single‐cell suspension had been seeded at 5000 cells/ml and incubated for 24 h to allow the attachment. Consequently, cells were treated with different concentrations of CSLME (10, 15, 20 μg/ml) and the medium was substituted with a fresh medium after 24 h of incubation and allowed them to grow for another 10 days. Post incubation, the colonies were fixed with 4% (wt/vol) paraformaldehyde for 20 min under room temperature and stained with 0.1% (wt/vol) crystal violet for 10 min. The six well plate was photographed and the number of colonies were counted. The colony formation percentage was determined with a formula,

$$\text{Colony formation potential}\;\left(\%\right)=\text{Number of colonies in treated}/\text{Number of colonies in control}\times 100$$

### 2.8 Detection of apoptosis using Hoechst 33258 staining

The morphological evaluation of apoptotic cells was assessed by nuclear staining using Hoechst 33258 staining dye (Sigma‐Aldrich, #B2261). The test was conducted using the previously described method.^25^ Briefly, the MCF‐7 cells were seeded at a density of 5 × 10^5^ in 12 well plates and permitted to reach 70% confluence and is further treated using the IC_50_ concentration of the CSLME (20 μg/ml) for 48 h at 37°C. Thereafter, the cells have been washed with PBS and stained with Hoechst 33258 solution (10 μg/ml) and afterward stained with propidium iodide (PI) and fixed using 4% formaldehyde solution. The stained cells were further washed with PBS and finally stained nuclei and morphological changes were imaged by a fluorescence microscope (Leica SP5 confocal microscope, Wetzlar, Germany).

### 2.9 Detection of DNA damage by DNA fragmentation assay

The assay was conducted according to the process mentioned previously.^26^ MCF‐7 cells (2 × 10^6^ cells/well/2 ml) have been grown in 6 well plates for 24 h with various CSLME concentrations. Cells were centrifuged for 10 min at 1500 rpm and washed with PBS after treatment. This pellet is then lysed using a lysis buffer (250 μl) with 5% Triton X‐100, 100 mM NaCl, pH 8.0, 10 mM Tris–HCl, 5 mM EDTA consisting of proteinase‐K (200 μg/ml) and incubated at 50°C for 1 h followed by 90‐min incubation with 400 μg/ml DNase‐free RNase. The extraction of DNA was carried out in 100 μl of phenol:chloroform:isoamylalcohol in the ratio of 25:24:1 and the mixture was centrifuged. DNA from the aqueous phase has been precipitated with chilled alcohol (3 ml) and sodium acetate (0.3 M) at 20°C overnight. Centrifugation of the precipitate was carried out at 13 000 × 25 *g* for 10 min. Further, the DNA pellet was then washed with alcohol (80%), followed by drying and then dissolved in a 50 μl Tris EDTA buffer, mixed in loading buffer and 1% agarose gel was used for electrophoresing up to 1.5 h, at 80 V in TAE buffer (Tris‐acetate EDTA).

### 2.10 Confirmation and quantification of CSLME induced apoptosis using Annexin V FITC/PI dual staining assay

CSLME mediated apoptosis of MCF‐7 cells was calculated by using the slight changes in the method mentioned earlier^27^ using Annexin V‐fluorescein isothiocyanate (FITC)/PI kit. Post the treatment with CSLME (20 μg/ml) for 48 h, MCF‐7 cells have been collected and washed with cold PBS twice and resuspended in 400 μl 1X binding buffer. Further, the cell suspension was mixed with 2 μl of Annexin V‐FITC antibody and the cells were incubated for 30 min at 37°C. Immediately before flow cytometric analysis, PI (10 μl) was added. PI was used to identify cells that have lost the integrity of their membrane. Data was acquired using BD FACS Calibur.

### 2.11 Effect of CSLME on F‐actin cytoskeleton organization

To understand the effect of CSLME on F‐actin cytoskeleton derangement, MCF‐7 cells were seeded and incubated for 24 h at a density of 2 × 10^5^ on a coverslip in 12 well plate. The cells were either treated with VC or CSLME (20 μg/ml) for 24 h. Post the treatment, the actin filaments were stained with phalloidin‐FITC, while the nuclei were stained with DAPI. The captured images were taken by Leica confocal (Leica SP5 confocal microscope) microscope and a merged image profile of phalloidin‐FITC and DAPI stained cells was analyzed with LAS AF lite software.

### 2.12 Cell cycle analysis

CSLME induced changes in cell cycle was analyzed according to a previously described method.^28^ MCF‐7 cells were seeded in 6 well plates at a density of 5 × 10^5^ cells/well and treated with CSLME (20 μg/ml) for 24 h. These cells are then washed and centrifuged at 1800 rpm for 8 min. After the treatment, the cells were re‐suspended and fixed at 4°C with 70% ethanol for 2 h. After fixation the cells were washed with PBS and centrifuged for 8 min at 1800 rpm. The pellet was then dispersed and re‐suspended into 250 μl PBS, PI (20 μg/ml; ThermoFisher Scientific, # BMS500PI), and RNase A (20 μg/ml; Sigma‐Aldrich, #R4875) and incubated for 30 min in the dark. Finally, the cells were analyzed by flow cytometry (BD FACs Calibur, BD Biosciences, United States).

### 2.13 Effect of CSLME on angiogenesis: CAM assay

The chorioallantoic membrane (CAM) test was carried out according to our previous study.^29^ Anti‐angiogenic potential (percent) was represented by a formula, 1 − *T*/*C* × 100, where *T* represents the number of blood vessels crossing a CSLME‐treated disc, and *C* shows the number of blood vessels crossing the disc in the control experiment.

### 2.14 Western blot analysis of anti and pro‐apoptotic markers, and proinflammatory transcription factor NF‐κB

To understand the mechanistic pathway of apoptosis triggered by CSLME, the expression profile of anti‐apoptotic marker (Bcl2), pro‐apoptotic markers (Bad, Bax), and apoptosis mediating enzymes such as Caspase‐9 and PARP were analyzed using western blot analysis (WBA) as per our previous methodology.^3^ In brief, 5 × 10^5^ MCF‐7 cells were seeded in cell culture dishes and incubated for 24 h. These cells were then treated with the CSLME (20 μg/ml) in a humidified CO_2_ incubator (5% CO_2_) at 37°C up to 72 h. The cells were then scraped, pelleted, and lysed with a RIPA buffer. The resulting cell lysate was centrifuged for 20 min at 14 000 rpm at 4°C. Protein quantification of samples was performed with the help of the Bradford Protein Estimation System (BioRad) and the equal amount of protein (50 μg) was resolved on 8%–12% SDS‐PAGE. Sea Blue Protein Ladder (Invitrogen) was used as a molecular weight marker.

The SDS resolved proteins were further transferred onto PVDF membranes (Santa Cruz) at 200 mA for 6 h at 4°C. The protein‐adsorbed membranes are incubated in the blocking buffer (comprising of with 5% dry milk [non‐fat] in tris buffered saline, 150 mM NaCl and 10 mM Tris pH 7.5) for 1 h, followed by overnight incubation with the primary antibodies (against Bax (1:1000; sc‐7480, Santa Cruz), Bad (1:1000; sc‐8044, Santa Cruz), Bcl2 (1:1000; sc‐7382, Santa Cruz), PARP‐1 (1:2000; 9532, Cell Signaling Technology), cleaved PARP‐1 (1:2000; 9548, Cell Signaling Technology), caspase‐9 (1:2000; 9502, Cell Signaling Technology), cleaved caspase‐9 (1:2000; 9509, Cell Signaling Technology), phospho NF‐κB p65 (ser468) (1:2000; 3039, Cell Signaling Technology), NF‐κB p65 (1:2000; 8242, Cell Signaling Technology, and Actin (1:1000; sc‐47 778, Santa Cruz) in blocking buffer at 4°C. Three times every 10 min, the PVDF membranes have been thoroughly washed with TBS‐T (TBS contacting 0.1% Tween‐20) and incubated using HRP tagged secondary antibodies (1:1000; sc‐2357, Santa Cruz**)** for 1 h at room temperature. The protein bands have been developed using the Amersham ECL Prime western blotting reagent and have been visualized using an enhanced chemiluminescence detection device (Bio‐Rad ChemiDoc XRS+). To understand the fold expression pattern of the blotted proteins, differences in the band intensities between control and CSLME treated MCF‐7 cells were measured by densitometry and represented in the form of fold change.

### 2.15 In vivo acute toxicity study of CSLME

The Organization for Economic Cooperation and Development (OECD) Guidelines 423 were used to perform an acute toxicity analysis. The research included male albino *Wistar* rats having weighed between 200 and 250 g. In Pinnacle Biomedical Research Institute (PBRI), Bhopal, India, the mice were housed at an animal house. All animal studies have been approved by Institutional Animal Ethics Committee (IAEC) of Pinnacle Biomedical Research Institute (PBRI), Bhopal (CPCSEA Reg. No. 1824/PO/ERE/S/15/CPCSEA; and protocol approval reference No. PBRI/IAEC/PN‐17022). One week before dosing, rats were acclimatized to laboratory conditions and were kept in polycarbonate cages with husk bedding in a group of three animals. All animals were kept at 22 ± 2°C in 12:12 h, light: dark period. Doses of 5, 50, 300, 2000 mg/kg body weight of CSLME were given consecutively by the oral route using sterile gavage. All animals were observed daily for all important signs. If symptoms are observed after the beginning, the intensity of the duration was recorded and mortality for all animals was observed every day for the duration of the trial. The lethal dose of 50% (LD_50_) was estimated as per the method depicted by OECD Guidelines 423.

### 2.16 In vivo anti‐breast cancer activity of CSLME by using xenograft animal model study

For assessing the in vivo anti‐tumor effect of CSLME, the female NOD‐SCID mice (6–8 weeks old; *n* = 6 per group) were used for xenograft model studies. Experiments were performed in strict compliance with the principles of institutional ethics. The Institutional Review Board (IRB) of the ACTREC: “Advanced Center for Treatment Research and Education in Cancer,” Tata Memorial Hospital, Kharghar, Navi Mumbai, Maharashtra (India) was granted permission to conduct this research and ethics clearance. The animals were fed standard food and water ad libitum in laminar airflow cabinets under pathogen‐free conditions with a 12‐h dark/light period. MCF‐7 breast cancer cells (5 × 10^6^) were injected subcutaneously in 100 μl of PBS in the female NOD‐SCID mice.^30^ Total mice were separated into two treatment groups, and one control group with 6 animals in each group. After detecting visible xenografts, intraperitoneal injections of CSLME extract (25 mg/kg body weight) were administrated for 17 consecutive days whereas similar volumes of PBS were given to the control group. Following inoculation, all mice were monitored regularly for tumor development, body weight and health. Before gavage feeding, the tumor size was calculated at each stage. Diameters of tumors have been determined regularly until the end. With calipers, the long (*D*) and short (*d*) diameters were measured. The volume of tumors (cm^3^) was reported to be *V* = 0.5 × *D* × *d*.^31^

### 2.17 Statistical analysis

All the assays and experimental protocols described here were performed in triplicate and wherever necessary, the results are presented as mean values of *n* = 3 or *n* = 6 (for animal studies) ± SD and the significance of the difference from the respective controls was assayed by using Student's *t* test. *p* value of **p < .05 versus control was considered as a level of significance in respective assays/methods.

## 3 RESULTS

### 3.1 Qualitative and quantitative screening of CSLME

Table 1 summarizes the findings of CSLME's qualitative and quantitative phytochemical study. The qualitative analysis revealed the presence of alkaloid, tannin, terpenoid, and absence of saponin. The quantitative evaluation showed a considerable amount of phenol and flavonoid content. For example, in CSLME the phenol content has been predicted using gallic acid as standard and exhibited as gallic acid equivalents per gram of dry weight of extract (mg GAE/g DW). CSLME was also found to have a significant phenol amount (79.26 mg GAE/g DW). The flavonoid content in CSLME was calculated with catechin as standard and was shown to be catechin equivalents per gram of dry extract weight (mg CAE/g DW). An amount of 43.63 mg of CAE/g DW of total flavonoid was estimated in plant extract (Table 1).

**TABLE 1:** Qualitative and quantitative phytochemical analysis of CSLME

Test sample	Qualitative screening				Quantitative estimation	
	Alkaloid	Tannin	Terpenoid	Saponin	Phenolics (mg) GAE/g DW	Flavonoid (mg) CAE/g DW
*Chloroxylon swietenia* (CSLME)	+	+	+	−	79.26 ± 1.17	43.63 ± 1.20

*Note*: For quantitative test, the numerical results are expressed as the mean values from three independent experiments ± SD. The total phenolics was estimated as mg GAE/gDW = gallic acid equivalents per gram of dry weight of extract, while the total flavonoid was estimated as mg CAE/gDW = catechin equivalents per gram of dry weight of extract. **+** indicates presence of metabolites; − indicates absence of metabolites.

### 3.2 GC–MS analysis of CSLME

The results of the GC–MS chemoprofile of the CSLME are summarized in Table 2. Variety of structurally diverse phytochemicals such as, 1‐(4‐methoxyphenyl)‐2,5‐dimethyl‐1H‐pyrrole‐3‐carbaldehyde; 4‐methoxy‐7H‐furo[3,2‐g]chromen‐7‐one; 7‐methoxy‐8‐(3‐methylbut‐2‐en‐1‐yl)‐2H‐chromen‐2‐one; 1,4‐di‐tert‐pentylbenzene; 7‐methoxy‐6‐(3‐methyl‐2‐oxobutyl)‐2H‐chromen‐2‐one; 4,7,8‐trimethoxyfuro[2,3‐b]quinoline; (E)‐5‐methyl‐1‐(2,6,6‐trimethylcyclohexa‐2,4‐dien‐1‐yl)hexa‐1,4‐dien‐3‐one; (E)‐3,7,11,15‐tetramethylhexadec‐2‐en‐1‐ol; 4‐hydroxy‐9‐(3‐methylbut‐2‐en‐1‐yl)‐7H‐furo[3,2‐g]chromen‐7‐one; 1‐[(2‐bromophenoxy)methyl]‐3,4‐dimethoxy; 9,12,15,‐Octadecatrioenoic acid methyl ester and Benzene, *N*‐hexadecanoicacid, and so forth were observed as major phytoconstituents in CSLME.

**TABLE 2:** Phyto components identified in CSLME by using GC–MS analysis

No.	Name of the compound	Structure of phytochemical	Molecular formula	Molecular weight	Retention time (min)
1	4‐methoxy‐7H‐furo[3,2‐g]chromen‐7‐one	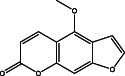	C_12_H_8_O_4_	216.04	23.15
2.	(7‐methoxy‐8‐(3‐methylbut‐2‐en‐1‐yl)‐2H‐chromen‐2‐one)	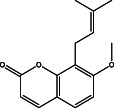	C_15_H_16_O_3_	244	25.61
3	1‐(4‐methoxyphenyl)‐2,5‐dimethyl‐1H‐pyrrole‐3‐carbaldehyde	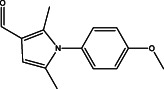	C_14_H_15_NO_2_	229.11	25.95
4	1,4‐di‐tert‐pentylbenzene	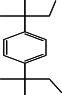	C_16_H_16_	218	26.76
5	7‐methoxy‐6‐(3‐methyl‐2‐oxobutyl)‐2H‐chromen‐2‐one	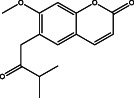	C_15_H_16_O_4_	260	27.45
6	4,7,8‐trimethoxyfuro[2,3‐b]quinoline	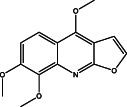	C_14_H_13_NO_4_	259	27.83
7	(E)‐5‐methyl‐1‐(2,6,6‐trimethylcyclohexa‐2,4‐dien‐1‐yl)hexa‐1,4‐dien‐3‐one	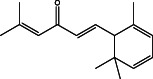	C_16_H_22_O	230	29.07
8	4‐hydroxy‐9‐(3‐methylbut‐2‐en‐1‐yl)‐7H‐furo[3,2‐g]chromen‐7‐one	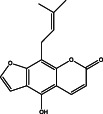	C_16_H_14_O_4_	270	30.16
9	(E)‐3,7,11,15‐tetramethylhexadec‐2‐en‐1‐ol	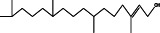	C_20_H_40_O	296	17.62
10	N‐hexadecanoic acid	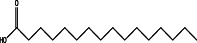	C_16_H_32_O_2_	256	20.14
11	9,12,15,‐Octadecatrioenoic acid methyl ester	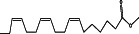	C_19_H_32_O_2_	292	24.34
12	1‐[(2‐bromophenoxy)methyl]‐3,4‐dimethoxy	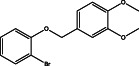	C_15_H_15_Br O_3_	323	28.72

### 3.3 MTT assay

**FIGURE 1:**
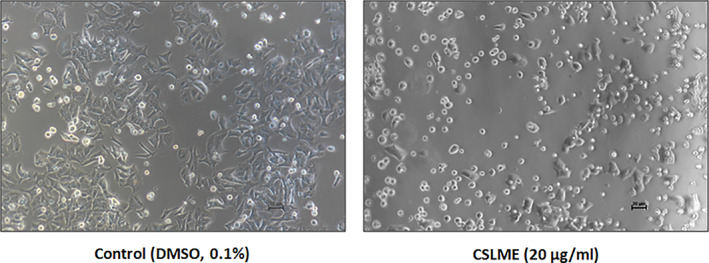
Representative images of MCF‐7 cells treated with either 0.1% of DMSO (vehicle control) or 20 μg/ml of *Chloroxylon swietenia* leaves methanol extract (CSLME) after 72 h of treatment. The adverse effects of CSLME treatment on viability and morphology can be apparently compared with control cells

In‐vitro cytotoxicity activity of CSLME against MCF‐7 was determined using MTT assay. The findings are given as IC_50_ values. The reference compound was Adriamycin. The microscopic image depicted in Figure 1, clearly shows the adverse effects of CSLME on the cellular morphology of MCF‐7 cells. The findings have shown CSLME has promising cytotoxic activity against MCF‐7 cells with an IC_50_ value of 20 μg/ml compared with Adriamycin (positive control, IC_50_, 3 μg/ml).

### 3.4 Effect of CSLME on cell migration (Scratch and transmigration assay)

**FIGURE 2:**
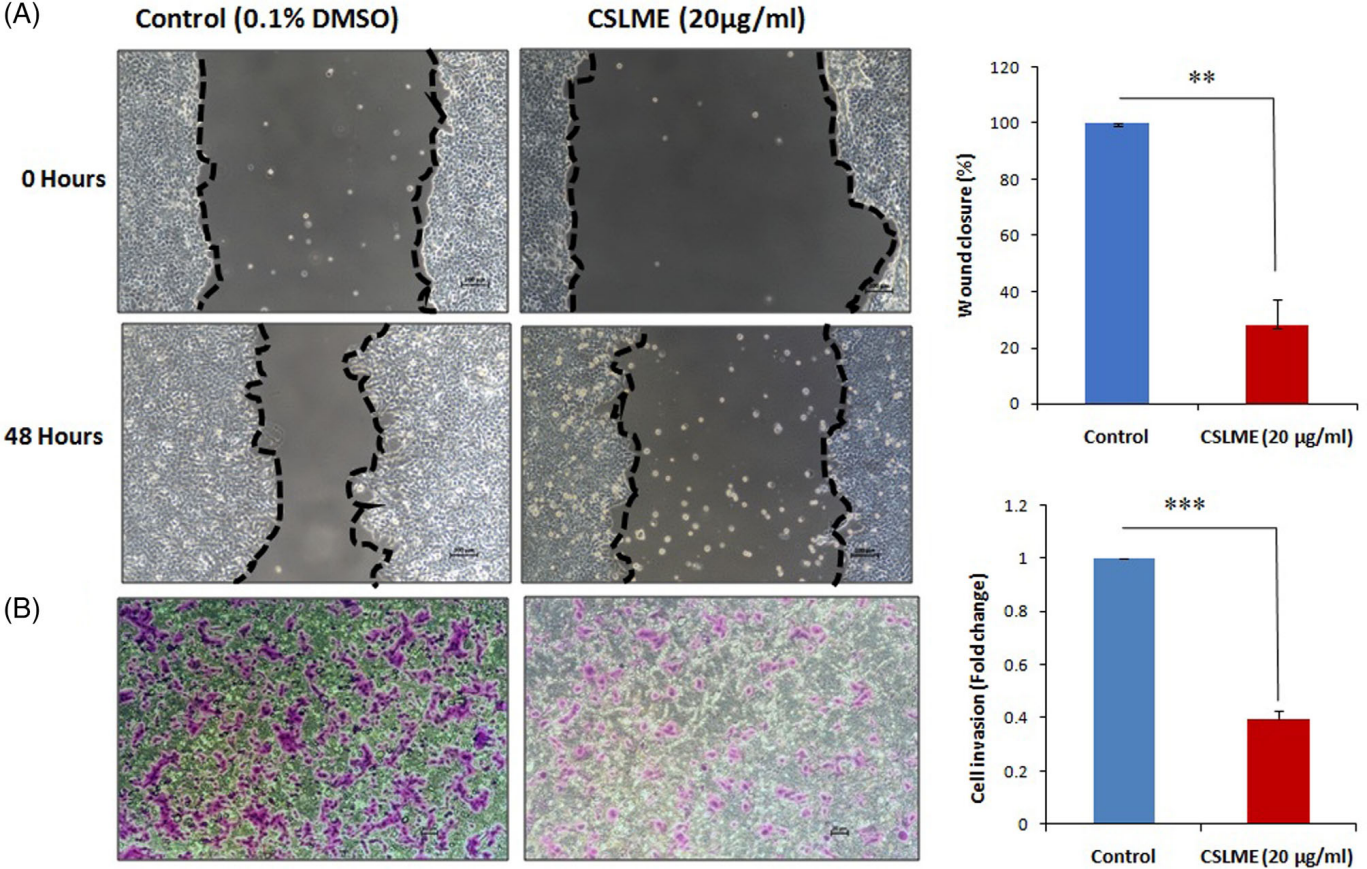
Effect of *Chloroxylon swietenia* leaves methanol extract (CSLME) on migration of MCF‐7 cells. (A) MCF‐7 cells were cultures in monolayer, wound scratch was generated using 10 μl pipette tip and wound closure was observed for 24 h. Images were captured at 0 and 48 h. The area of wound closure were measured, percentage of wound closure was calculated and represented in the form of bar graph. (B) MCF‐7 cells were treated with either vehicle control (0.1% DMSO) or 20 μg/ml of CSMLE and seeded on the upper chamber of transwell inserts. DMEM with 10% FBS was used as a chemoattractant and cells were allowed to migrate toward the lower chamber. Number of migrated cells was counted and difference in the number of migrated cells was represented as bar graph. ***p < .05 versus control

To study the effect of CSLME in the regulation of breast cancer cell migration, both wound scratch assay and transwell migration assay was performed on MCF‐7 cells (Figure 2). In both the assays, control or untreated cells have migrated to a greater extent, whereas there is an apparent reduction in the cell motility when the cells were treated with CSLME (20 μg/ml). The results clearly demonstrate that CSLME inhibited the migration capability of MCF‐7 cells.

### 3.5 Effect of CSLME on colony formation (clonogenic assay)

**FIGURE 3:**
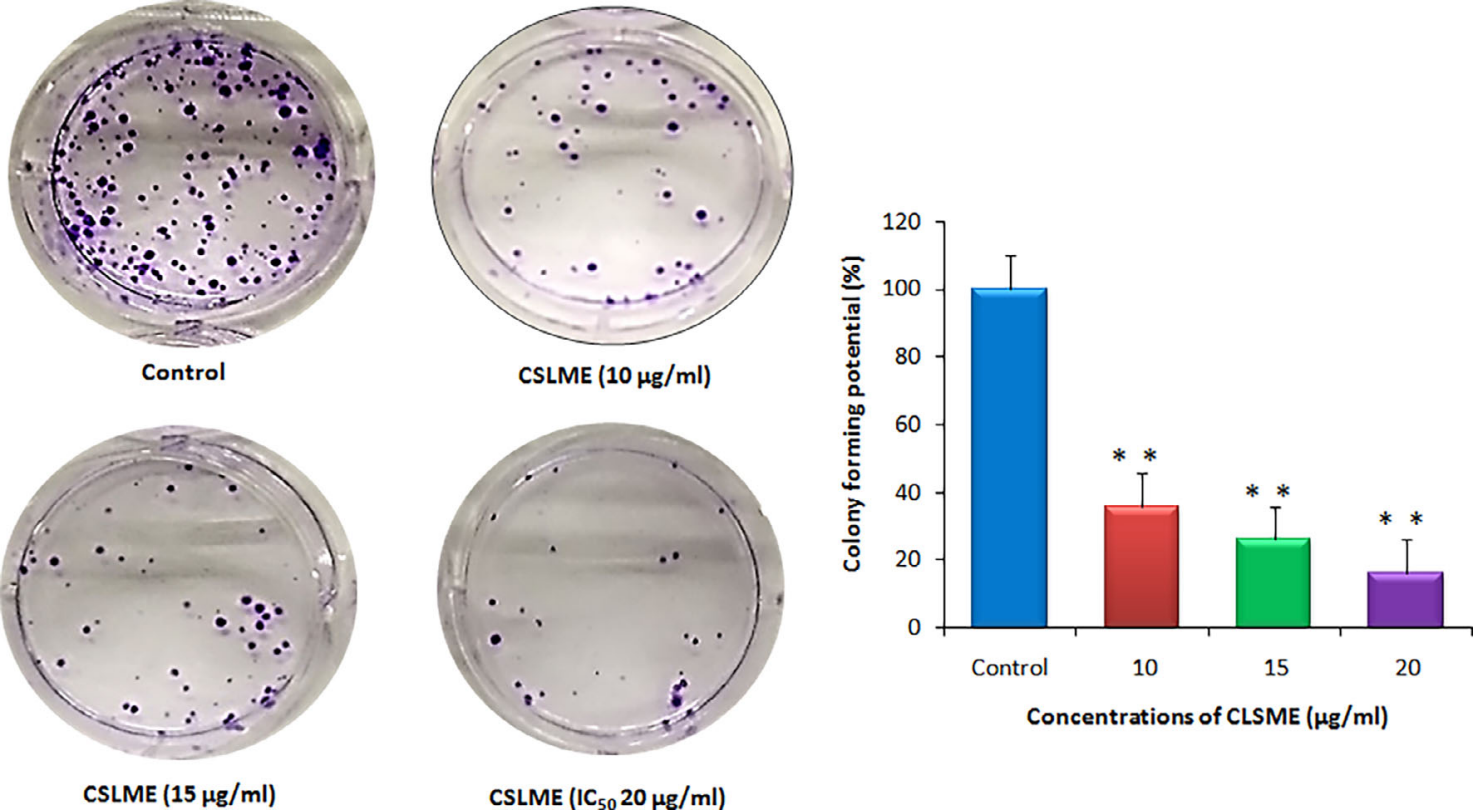
Effect of *Chloroxylon swietenia* leaves methanol extract (CSLME) on colony formation ability of MCF‐7 cells. Representative images of the clonogenic assay showing the effect of CSLME (10, 15, and 20 μg/ml) on colony formation ability of MCF‐7 cells. MCF‐7 cells were seeded at low density in six well plates and allowed to form colonies. The cells were fixed, stained by crystal violet and number of colonies were counted using colony counter. The data represents percent colonies formed in treated samples compared to untreated cells. A significant reduction in the number of colonies 36%, 26.5%, and 16.9% were observed in MCF‐7 cells treated with 10, 15, 20 μg/ml of CSLME respectively. **p < .05 versus control. The data presented is the mean value of *n* = 3 ± SD

To determine the effect of CSLME extract in regulating the cell survival of breast cancer cells, colony formation assay was performed on MCF‐7 cells (Figure 3). In this study, cells were treated with different concentrations of CSLME (10, 15, and, 20 μg/ml) and results are presented in Figure 3. The concentrations 10 and 15 μg/ml, which are lower than IC_50_ (20 μg/ml) concentration were selected to rule out the possibility that colony inhibition is not related to cell death. As compared to control (100%), treatment with 10, 15, 20 μg/ml of CSLME demonstrated 36%, 26%, and 16% colony formation potential respectively. These results demonstrate that CSLME reduced the colony‐forming potential of MCF‐7 cells, which has been considered as an important aspect of cancer chemotherapy drugs.

### 3.6 Detection of apoptosis using Hoechst 33258 staining

**FIGURE 4:**
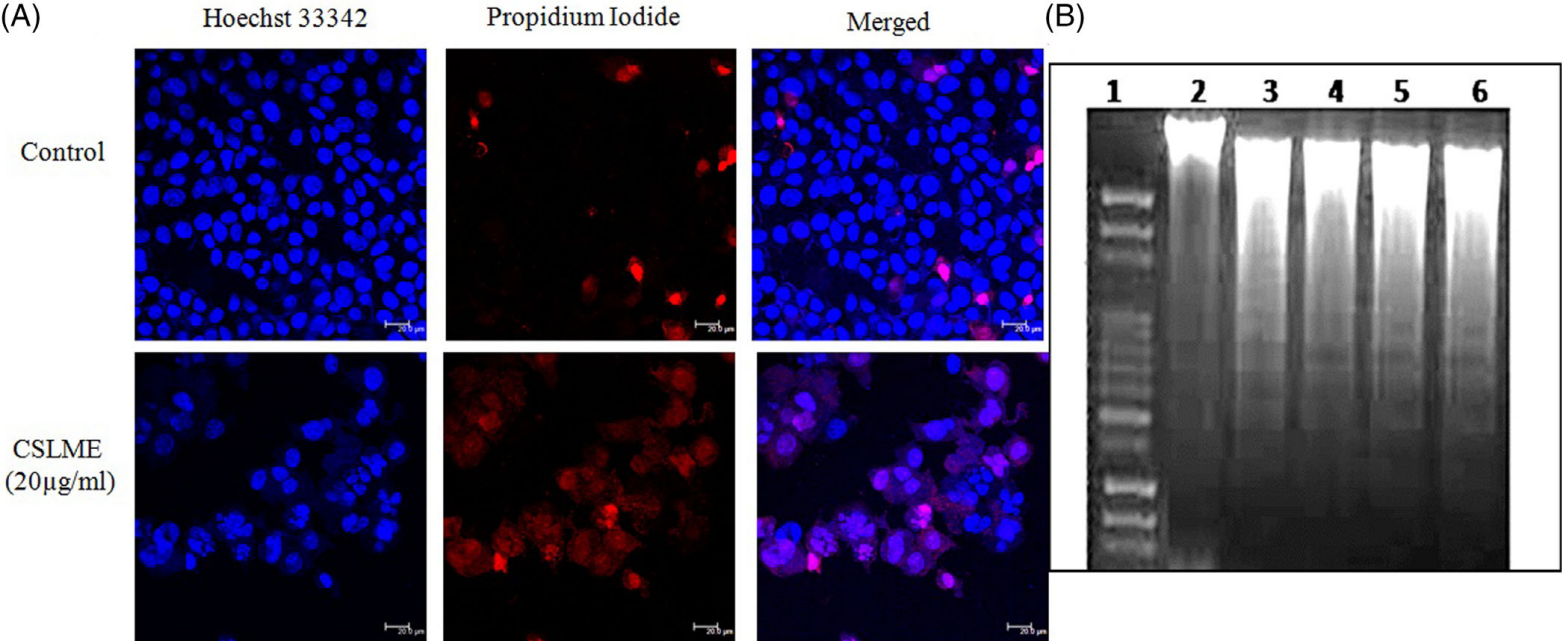
(A) Representative confocal microscopy images of MCF‐7 cells stained using Hoechst 33258 nuclear stain along with propidium iodide and captured at 63× magnification. Upper panel showing control MCF‐7 cells (DMSO 0.1%) and Lower panel MCF‐7 cells treated with *Chloroxylon swietenia* leaves methanol extract (CSLME) (20 μg/ml) showing apoptotic fragmented nuclear morphology. (B) Agarose gel image showing DNA fragmentation in MCF‐7 cells induced by CSLME treatment. DNA extracted from control and different concentration of CSLME (10, 20, 30, and 40 μg/ml) treated cells was resolved on 1% agarose gel by electrophoresis. Lane 1‐ Marker, lane 2‐ control, lane 3–10 μg/ml, lane 4–20 μg/ml, lane 5–30 μg/ml, and lane 6–40 μg/ml of CSLME treated MCF‐7 cells respectively

After 48 h of treatment, many irregular biological changes and adverse effects were observed in the cell membrane and nucleus of CSLME treated MCF‐7 cells. The nucleus of untreated control cells typically appeared blue with no distinctive pattern of cellular morphological changes (Figure 4). On the other hand, the cells treated with CSLME were seen with nuclear change and apoptotic body developments and characterized by nuclear fragmentation, chromatin condensation, nuclear shrinkage, and cytoplasmic blebbing, and so forth which otherwise represents the morphological signatures of cells undergoing apoptosis. These cytological changes have indicated strongly that the cells have dedicated themselves to apoptotic cell death when treated with CSLME.

### 3.7 Detection of DNA damage by DNA fragmentation assay

The CSLME induced apoptosis induction was further confirmed by determining the DNA fragmentation patterns. DNA fragmentation of MCF‐7 treated cells was identified on 1% agarose gel electrophoresis. After treatment with different CSLME concentrations for 24 h, DNA fragmentation was examined. The CSLME‐treated cells displayed DNA laddering characteristics which are a distinctive feature of apoptosis, while control cells did not show the ladder pattern, as shown in Figure 4. Present result proved that apoptosis was followed by DNA fragmentation in MCF‐7 cells after exposure to CSLME. DNA fragmentation is one of the depictive biochemical signatures of apoptosis.

### 3.8 Confirmation and quantification of CSLME induced apoptosis using Annexin V FITC/PI dual staining assay

**FIGURE 5:**
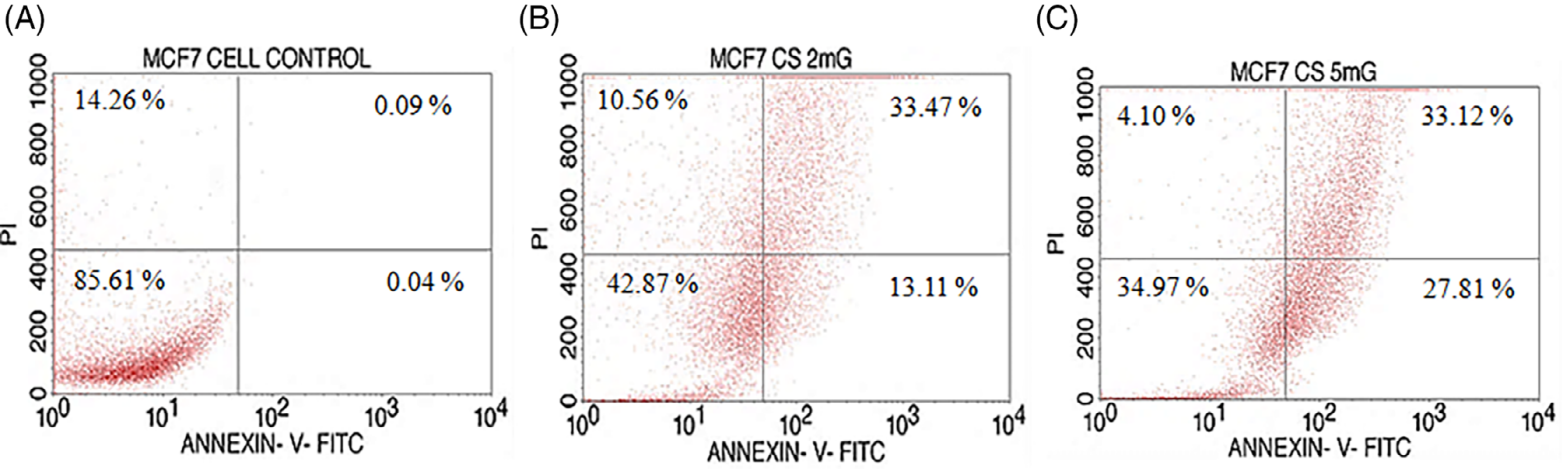
Confirmation of *Chloroxylon swietenia* leaves methanol extract (CSLME) induced early apoptosis using Annexin‐V/PI assay. (A–C) MCF‐7 cells were treated with different concentrations of CSLME (2 mg and 5 mg/ml) and vehicle control (DMSO 0.1%) for 48 h. The cells were subjected to Annexin‐V/PI staining, percentage of apoptotic population was analyzed by using flowcytometry

Flow cytometry analysis was performed to examine and quantify the proportion of breast cancer cells going through apoptosis by using Annexin‐V conjugated with FITC/PI detection kit. MCF‐7 cells have been stained Annexin V‐FITC with or without PI, after‐treatment of the different concentrations of CSLME over 48 h. The flow cytometry analysis of the control/untreated cells showed that the cells were primarily negative for Annexin V‐FITC and PI staining showing they were viable and not undergoing apoptosis as shown in Figure 5. As illustrated in Figure 5, after 48 h of treatment, concentration dependent increase in the proportion of early and late apoptotic MCF‐7 cells were observed with CSLME at 2 mg/ml (46.58%) and at 5 mg/ml (60.93%). The late apoptosis cell population is usually high due to cell fragmentation leading to several apoptotic bodies with intact cell membranes containing cytoplasmic organelles and with or without nuclear fragments.^32^ The present results demonstrated that the CSLME induces the breast cancer cell death by means of early apoptosis.

### 3.9 Effect of CSLME on F‐actin cytoskeleton organization

**FIGURE 6:**
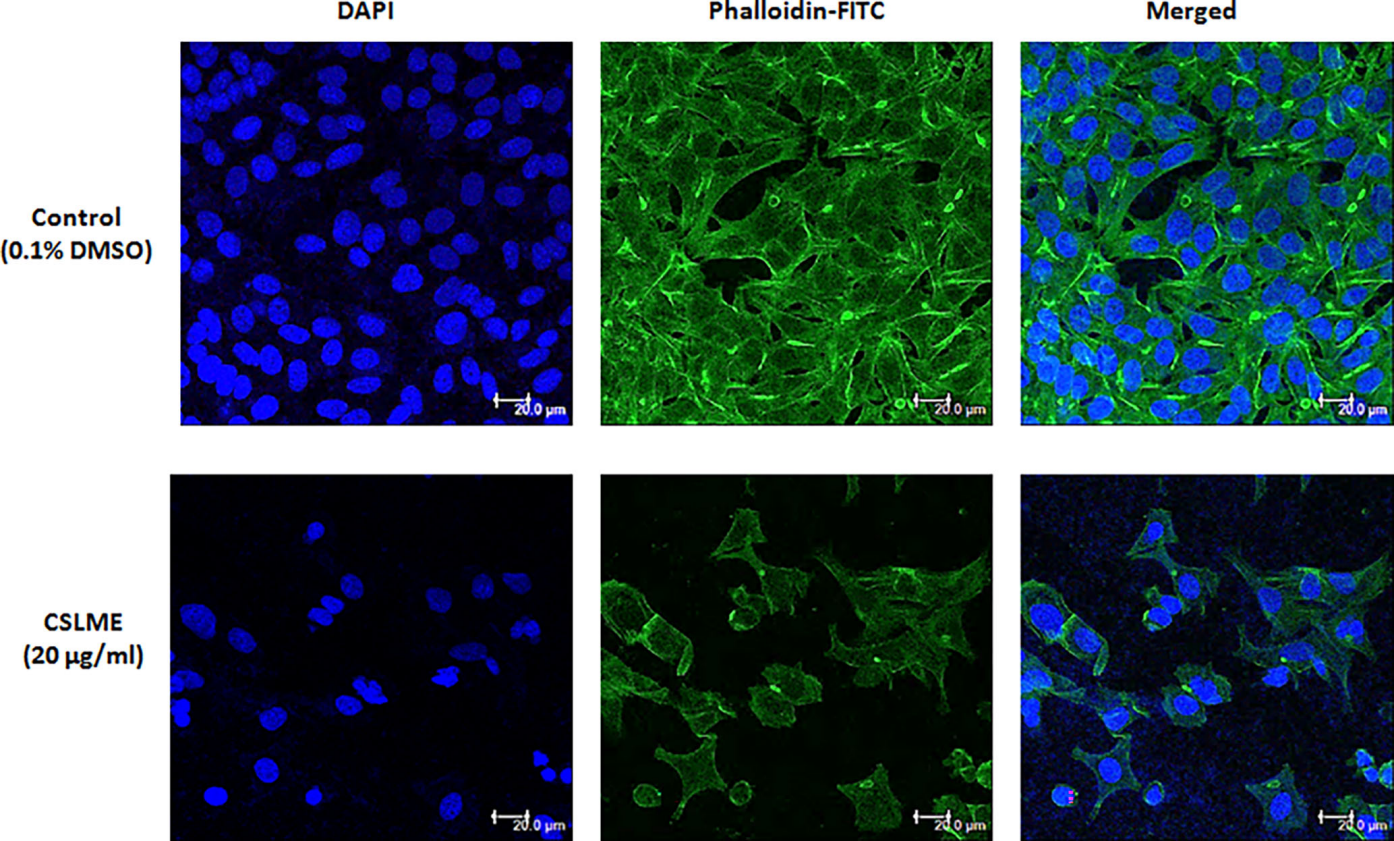
Effect of *Chloroxylon swietenia* leaves methanol extract (CSLME) treatment on the actin cytoskeleton disruption in MCF‐7 cells. MCF‐7 cells were incubated with either DMSO (vehicle control) or CSLME (20 μg/ml). After treatment, the actin filaments were then stained with phalloidin‐FITC, while the nuclei were stained with DAPI. The DAPI and FITC stained images were captured using a fluorescence microscope and a merged profile was generated. Scale bar, 20 μm; magnification 400×

The digitized results of MCF‐7 cells treated with CSLME followed by staining with phalloidin‐FITC (for actin) and DAPI (for nucleus) are shown in Figure 6. The DAPI and phalloidin‐FITC merged image profile clearly showed the disorganized architecture of actin microfilaments and nuclei in CSLME treated cells, however the nuclear and F‐actin cytoskeleton organization in the control set was observed to be normal. Thus, these results demonstrated that actin filament disorganization is associated with CSLME‐induced apoptosis in MCF‐7 cells.

### 3.10 Cell cycle analysis

**FIGURE 7:**
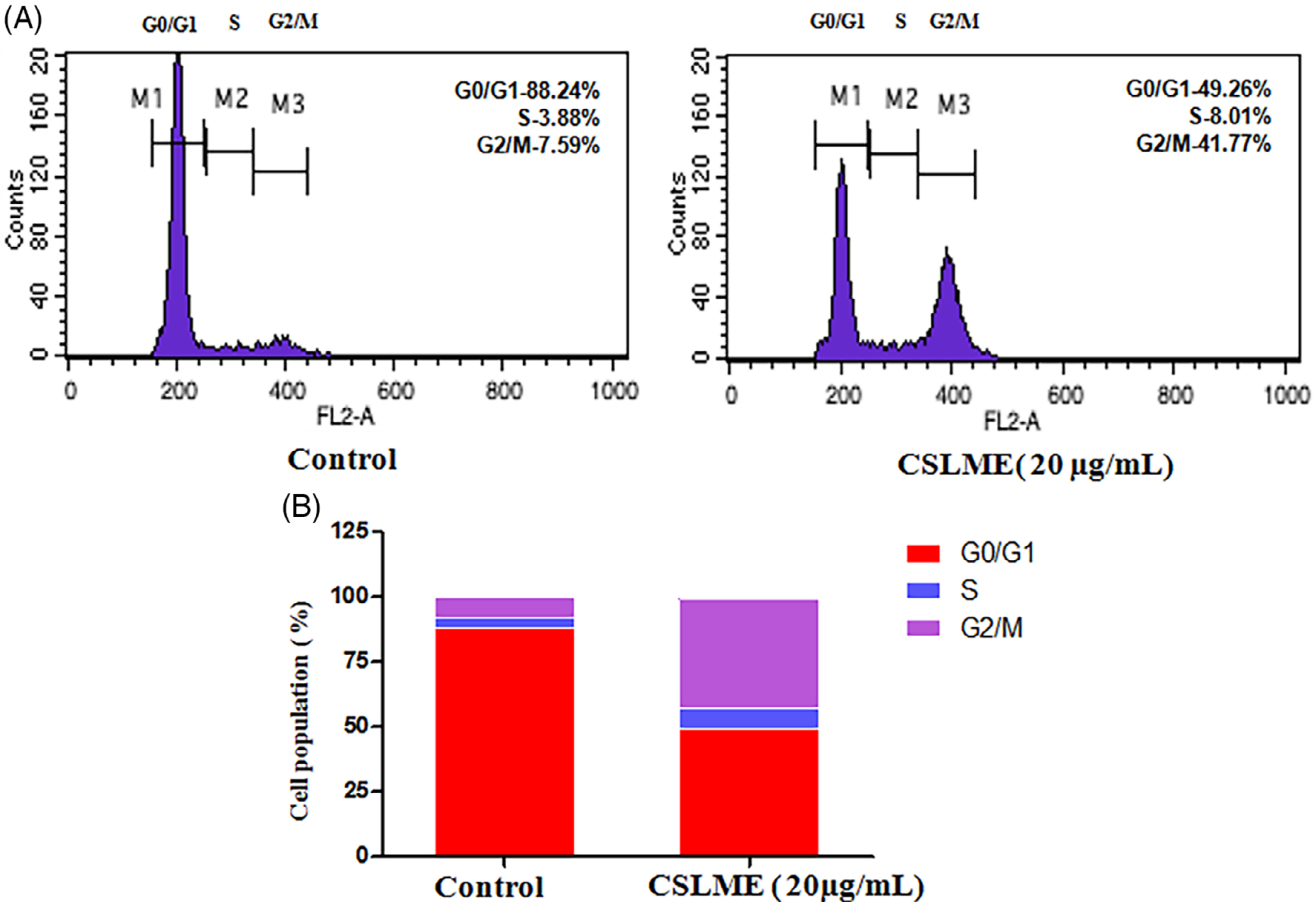
Induction of G2/M phase cell cycle arrest by *Chloroxylon swietenia* leaves methanol extract (CSLME) in MCF‐7 cells. MCF‐7 cells were incubated with either DMSO (vehicle control) or CSLME (20 μg/ml) and cell cycle distribution was analyzed using flowcytometry. (A) Histogram of cell cycle phase distribution of Control and CSLME (20 μg/ml) treated MCF‐7 cells. (B) Quantitative representation of cell cycle phases

This analysis is done to understand whether apoptotic cell death is due to the CSLME's inhibitory effect on the cell cycle processor not. The MCF‐7 cells were treated with the CSLME (20 μg/ml) for 24 h, stained with PI and then evaluated with flow cytometry. It is evidently shown that after CSLME treatment, cell cycle arrest was observed at G2/M phase (Figure 7).

### 3.11 Effect of CSLME on angiogenesis: CAM assay

**FIGURE 8:**
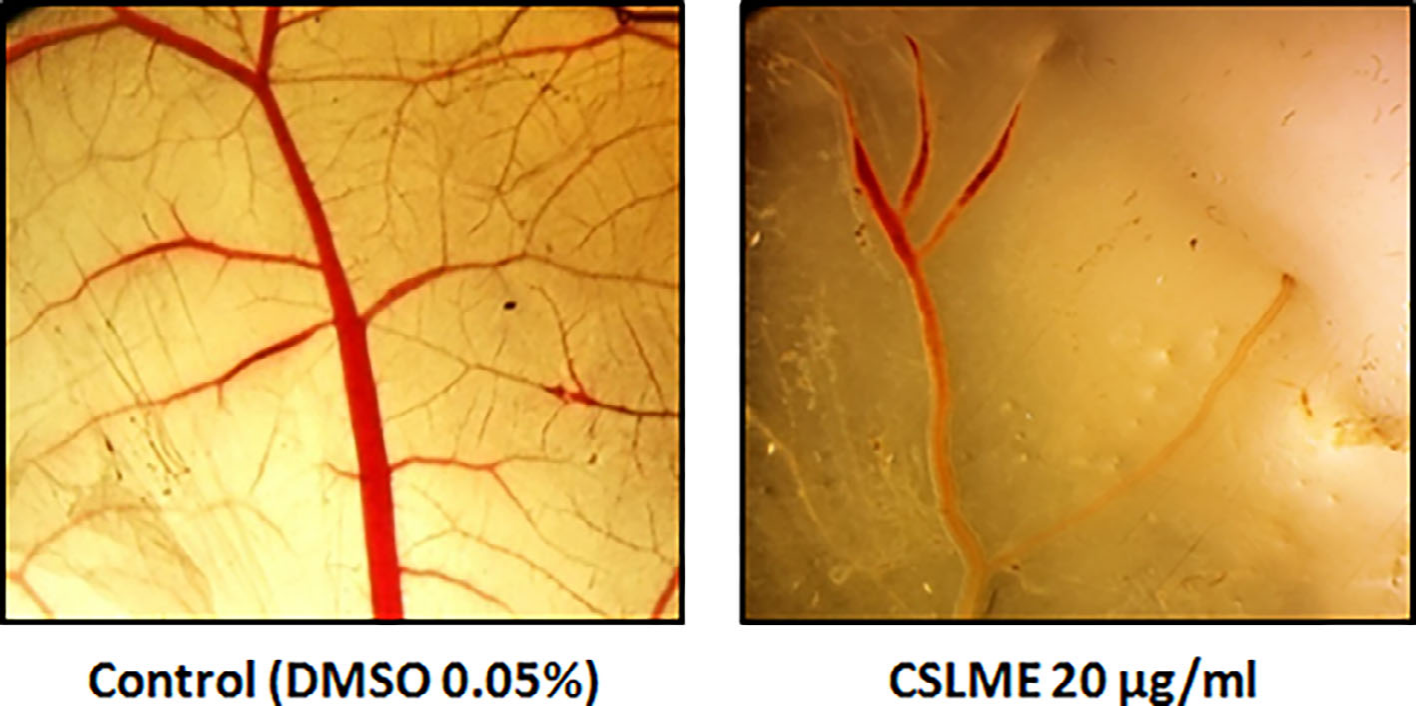
Inhibition of blood vessel formation by *Chloroxylon swietenia* leaves methanol extract (CSLME). Representative digitized illustrations of the chorioallantoic membranes (CAMs) exposed to IC50 concentration of CSLME (20 μg/ml). The images of CAMs were digitized using Olympus make SZ61TR Zoom Trinocular Microscope with CCD attached camera and an image capturing software Pinnacle v. 6.0.2 (build 152)

The anti‐angiogenic ability of CSLME extract at IC_50_ concentration was assessed by using the CAM assay in chick eggs. CSLME (89.90 ± 0.56%) displayed a promising antiangiogenic activity which is demonstrated by reduced vascularization of CAMs that are exposed to CSLME as compared to the control set (Figure 8). Digitized images showing CAM vascularization were subjected to AngioQuant v 1.33 (MATLAB‐based angiogenesis quantifying software) for the examination of length, number, size, and the number of junctions of the tubule complexes. The findings showed that the length, number, size, and number of junctions of tubular complexes gradually declined.

### 3.12 WBA of pro and anti‐apoptotic markers and pro‐inflammatory transcription factor NF‐κB

**FIGURE 9:**
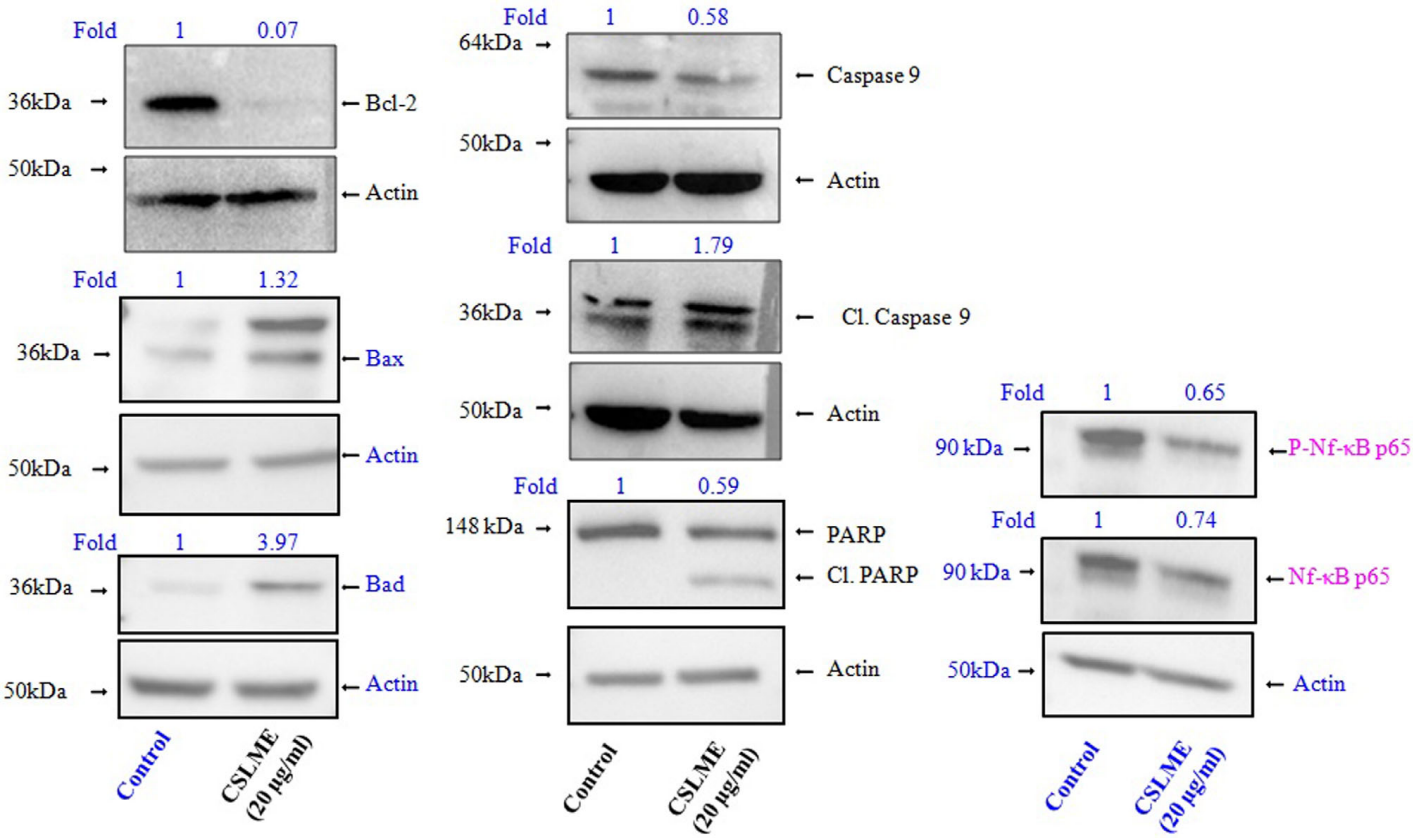
Effects of *Chloroxylon swietenia* leaves methanol extract (CSLME) in regulation of apoptosis related genes and Nf‐κB pathway in MCF‐7 cells. Western blot analysis of anti–apoptotic protein Bcl‐2, pro‐apoptotic proteins such as Bax and Bad, apoptosis‐related enzymes such as Caspase‐9, PARP and vital cellular pathway molecules, Nf‐κB p65 in control and CSLME (20 μg/ml) treated MCF‐7 cells. Differences in the band intensities between control and CSLME treated MCF‐7 cells were measured by densitometry and represented in the form of fold change

To understand the mechanistic pathway of apoptosis activated by CSLME in MCF‐7 cells, the immunoblotting of pro‐apoptotic proteins like Bax and Bad, and major anti‐apoptic protein such as Bcl2, and apoptosis‐related enzymes such as caspase‐9 and PARP‐1 was carried out. The results of immunoblotting presented in Figure 9, clearly showed the up‐regulation of Bax and Bad, while there was significant down regulation of anti‐apoptotic player like Bcl2. Further, it was observed that there was successful cleavage of caspase‐9 and PARP‐1 enzymes, which indicates the activation of intrinsic pathway in apoptotic cell death of MCF‐7 cells upon CSLME treatment.

To understand the molecular mechanism that regulates CSLME mediated anticancer activity, WBA of pro‐inflammatory transcription factor, NF‐κB was performed in CSLME treated MCF‐7 cells (Figure 9). Our data revealed that treatment of CSLME significantly down regulated the levels of p‐NF‐κB p65 in MCF‐7 cells compared to control. Our data also confirmed the down regulation total NF‐κB p65 in CSLME treated cells. These results suggest that CSLME might inhibit the viability of MCF‐7 cells through the down regulation of NF‐κB pathway.

### 3.13 In vivo acute toxicity study of CSLME

The acute toxic effect of CSLME was determined as per the OECD guideline 423, where the 2000 mg/kg of maximum test dose has been used. Three animals (*Albino wistar* rat) were used for each step. Fourteen days of study was performed, the study of acute toxicity measured general actions and lethality. CSLME was administered by oral gavage at a dose rate of 5 mg/kg body weight for Group I, 50 mg/kg body weight for Group II, 300 mg/kg body weight for Group III, and 2000 mg/kg body weight for Group IV. For the entire study period, all animals were monitored for mortality regularly. As there was no mortality observed even for the highest concentration of CSLME, that is, 2000 mg/kg body weight (Table 3). In all dose levels, no mortality and toxicity were observed in CSLME treated animals throughout the 14‐day dosing schedule. Therefore, the cut‐off dose (LD_50_) was concluded as 2000 mg/kg body weight (according to the adopted methodology).

**TABLE 3:** Assessment of in vivo acute toxicity of *Chloroxylon swietenia*

Group no.	Animals	Sex	Animals number	Dose (mg/kg body weight)	Lethality dead/tested
IA	AW Rat	Male	MC1 to MC3	5 mg/kg	0/3
IB	AW Rat	Male	MD1 to MD3	5 mg/kg	0/3
IIA	AW Rat	Male	MC1 to MC3	50 mg/kg	0/3
IIB	AW Rat	Male	MD1 to MD3	50 mg/kg	0/3
IIIA	AW Rat	Male	MC1 to MC3	300 mg/kg	0/3
IIIB	AW Rat	Male	MD1 to MD3	300 mg/kg	0/3
IVA	AW Rat	Male	MC1 to MC3	2000 mg/kg	0/3
IVB	AW Rat	Male	MD1 to MD3	2000 mg/kg	0/3

*Note*: No mortality was observed in all dose level group, hence LD_50_ cut off was concluded as 2000 mg/kg body weight as per adopted methodology.

### 3.14 In vivo anti‐breast cancer activity of CSLME by using xenograft animal model study

**FIGURE 10:**
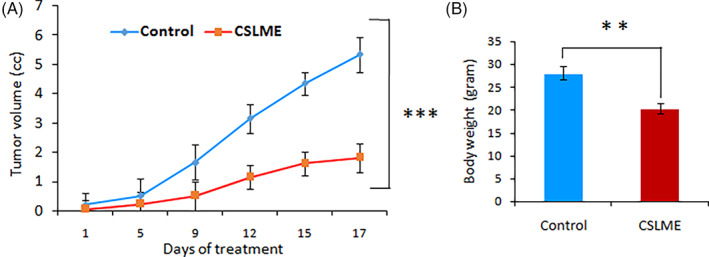
Inhibition of in vivo tumor growth by *Chloroxylon swietenia* leaves methanol extract (CSLME) in NOD‐SCID mice model (A) Line graph representing the tumor volumes in control and CSLME (20 mg/kg body weight) treated NOD‐SCID mice during the course of in vivo experiment. (B) Average body weights of the NOD‐SCID mice after administration of either PBS or CSLME. The results summarized are the mean values of *n* = 6 ± SD; **p < .05 versus control

To confirm the anti‐breast cancer activity of CSLME in vivo, the MCF‐7 xenograft NOD‐SCID mice model was used to perform the in vivo experiment. NOD‐SCID mice were inoculated subcutaneously with MCF‐7 cells and after 2 weeks of tumor development, the tumor‐bearing mice were treated intraperitoneally with 25 mg/kg of CSLME or PBS every day. After the 17th day of treatment, the development of the tumor and body weights of the mice were observed. The in vivo findings demonstrated treatment with CSLME considerably retarded tumor growth compared to treatment with PBS of mice carrying MCF‐7 tumors (Figure 10). After the trial (on the 17th day) the experimental group treated by the CSLME had substantially lower amounts of tumors compared to the control group.

## 4 DISCUSSION

Breast cancer is the most prominent cancer among females and influences around 1 in every 10 women worldwide, making it the second most predominant cancer type responsible for mortality in women.^33^ The drawbacks of the present chemotherapy treatments such as non‐specific toxicity, treatment resistance, and development of a secondary malignancy have motivated the examination of novel and efficient drugs against breast cancer. The work represents the evaluation of CSLME for their potential activity against breast cancer. The selection of plant was based on the extensive literature survey for their traditional uses and various reported biological activities.

The basic aim of the current investigation was to explore the potential of CSLME against MCF‐7 breast cancer cells using mechanistic approach. Initially, we evaluated CSLME's effect on both MDA‐MB‐231 (a triple‐negative cell line of breast cancer) and MCF‐7 (hormone‐dependent cell line of breast cancer). However, the sample was not significantly effective against MDA‐MB‐231, and therefore the studies were entirely focused on MCF‐7 cells. In this study, the CSLME displayed impressive cytotoxicity against MCF‐7 cells with 20 ± 0.11 μg/ml IC_50_ compared to Adriamycin reference compound (3 ± 0.49 μg/ml). The GC–MS chemoprofile of CSLME (Table 2) has revealed the diverse phytoconstituents belonging to phenols, alkaloids, flavonoids, and terpenoids which perhaps might be associated with their cytotoxicity and thereby demonstrating anti‐proliferative effect against MCF‐7 cells. The identified metabolites in CSLME like phenols, flavonoids, and terpenoids together with other heterocyclic compounds formulate the most pervasive groups of plant metabolites extensively reported for their anticancer effects.^34^ For example, the role of flavonoids in cancer hindrance specifies their essential effects on cancer chemoprevention as different flavonoids like flavanones, genistein, quercetin, daidzein, and luteolin are known for anticancer activity against different human cancers.^35^ Of note, the reputation of alkaloids as anticancer agents is also very wide as numerous previous reports suggested the anticancer effects of some currently prescribed alkaloids such as vinblastine, vincristine, and camptothecin are from plant origin.^36^

As described above the *C. swietenia* showed significant cytotoxicity against MCF‐7 cells and to the best of our knowledge, anti‐cancer and apoptosis inducing abilities of *C. swietenia* against cells of breast cancer has not yet been explored. As per the “US National Cancer Institute (US‐NCI) guidelines established for the evaluation of crude extracts, it has been advised that the crude extracts having IC_50_ concentration less than 100 μg/ml can be an active sample, moreover the crude samples with IC_50_ concentration less than 30 μg/ml may be labeled as a promising sample that can be further considered for purification of bioactive” leads.^37^ Therefore, we were inspired to set the objectives for further study to investigate the possible mechanisms of the anticancer effect of CSLME in MCF‐7 cells. To achieve the set objective, series of additional experiments were carried.

It is a well‐established fact that metastasis is one of the most important reasons for mortality in cancer. Metastasis is a multistep phenomenon involving cell adhesion, invasion, and migration. It is important to test the anti‐migration and invasion ability of anti‐cancer agents, as most of the current chemotherapy drugs are targeted toward the primary cancer cells and the metastasis targeting candidate drugs are yet to be evolved.^38^ Classically, the scratch or wound healing and the transwell migration assays are the model methodologies that mimic the invasion and migration abilities of the cancer cells under in vitro settings. The migration of cancer cells has been strongly implicated in cancer metastasis. In scratch or wound healing assay, the speed of wound closure and cell migration is measured with respect to the time, while in transmigration cell invasion assay, the potential of cell motility and invasiveness toward a gradient of chemo‐attractant is observed and calculated. Therefore, wound healing and transwell migration assays were conducted to investigate the impact of CSLME on invasion and migration potential of MCF‐7 cells. In both the assays, CSLME treated cells have demonstrated a substantial decrease in MCF‐7 cell invasion and migration potential (Figure 2). The results obtained indicate the anti‐metastatic potential of CSLME against MCF‐7 cells.

Clonogenic or colony formation assay is an in vitro cell survival assay based on a cell's ability to develop into a colony. The clonogenic assay mainly assesses the “unlimited” division ability of every cell in the population. This assay has remained a method of choice for the determination of cell reproductive death after treatment with the possible drug candidate. The clonogenic assay also states the ability of the cancer cell to proliferate and influence its regular morphological features when these cancer cells are treated with drugs. The failure of the replicative ability, loss of proliferation, and involvement of tumor suppressor genes are known to be associated with the inhibition of colony formation.^39^, ^40^ To examine further the colony inhibition potential of CSLME, a clonogenic assay was performed using three concentrations (10, 15, and 20 μg/ml). The results summarized in Figure 3, clearly demonstrated the colony inhibition potential of CSLME in MCF‐7 cells. The concentrations of 10 and 15 μg/ml, which are lower than IC_50_ (20 μg/ml) concentration of CSLME were deliberately selected to rule out the possibility that colony inhibition is not because of cell death alone, which per say is maximally induced at IC_50_ (20 μg/ml).

We investigated the apoptosis‐inducing potential of CSLME in MCF‐7 cells, as most of the phytochemicals are known to induce cell death by activating intrinsic or extrinsic apoptotic pathways in cancer cells. Series of scientific reports have described that the phytochemicals can effectively activate the apoptosis‐related cascade of marker proteins and enzymes and thereby induce cell death in a variety of cancer cells (reviewed by Kapinova et al.^41^). Apoptosis is a vital physiological system for the continuation of tissue homeostasis and plays a significant part in the pathogenesis of several disorders.

During apoptosis, some apparent morphological changes can be figured out as signatures of the initiation of the apoptotic process. In brief, the process starts with the condensation of chromatin in the nucleus, followed by nuclear fragmentation and ultimately the formation of apoptotic bodies. In several pathological situations, the disease is due to abnormal apoptosis while in others deficient apoptosis is the reason for disease progression. Cancer is one of the situations where deficient apoptosis is also a most important causative aspect in addition to uncontrolled cell proliferation which ultimately results in the survival of tumor cells. Apoptosis‐related cysteine proteases such as caspases and the members of the Bcl‐2 family of proteins including Bax, Bad, and Bcl‐2 are some of the key players in the process of cellular apoptosis. Hence, targeting the uncontrolled growth of malignant cells and deregulation of apoptosis are the evolving approaches for the design and development of anticancer agents.^32^

After assessing the cytotoxicity of CSLME, we were inspired to investigate the apoptosis‐inducing potential of CSLME and its mechanism in MCF‐7 cells, for which we have initially carried out the Hoechst 33258 nuclear staining test, which essentially helps to identify the nuclear morphology of cells undergoing apoptosis. The results summarized in Figure 4, confirmed that the nuclei of untreated breast cancer cells showed the intact nuclear structure whereas CSLME treated MCF‐7 cells showed distinctive morphological features of apoptosis such as condensed chromatin, cell shrinkage, cell nuclear fragmentation, and pyknotic (shrunken and dark) nuclei.^42^ Besides the morphological alteration of apoptosis in CSLME treated cells, DNA fragmentation of cells was also studied by assessing the formation of the DNA ladder. DNA fragmentation analysis of CSLME treated cells displayed a laddering pattern, which is one of the characteristic features of apoptosis.^43^

Furthermore, flow cytometry analysis of Annexin V and PI was performed to demonstrate the mechanism through which CSLME causes cytotoxicity in MCF‐7 cells. The presence of phosphatidylserine on the external leaflet of the cell membrane has been identified as one of the biomarkers of apoptosis.^12^ Anexin V‐FITC specifically binds to the phosphatidylserin residues and stains the apoptotic cells. PI is an intercalating agent used to stain nucleic acids of dead cells. In general, PI is impermeable to the cell membrane. However, dead cells contain a leaky membrane which allows the penetration of many dyes including PI. Hence, apoptotic cells show positive staining for both Annexin V and PI (Q2 in Figure 5). In contrast, necrotic cells show positive staining only for PI but nor Annexin V. The results summarized in the Figure 5 demonstrated the apoptotic‐inducing potential of the CSLME. The MCF‐7 breast cancer cell line showed an efficient CSLME sensitivity toward the apoptosis. The results of the Annexin V‐FITC and PI flow cytometry study also established the capability of the CSLME to induce the apoptosis in MCF‐7 cells but not the necrosis as there is no significant increase in the necrotic cell population (Q1) upon the treatment with CSLME (Figure 5). As the cancer cells demonstrate resistance to apoptosis to maintain their uncontrolled proliferation and therefore every apoptosis‐inducing agent is advantageous as a possible chemotherapeutic agent for the management of cancer progression.^12^

Because of variety of physiological functions, the intermediate microfilaments including actin have been considered as chemotherapeutic cancer targets for testing variety of anticancer agents including plant‐derived bioactive molecules. Owing to the clear pathophysiological link between F‐actin cytoskeleton and apoptosis, the effect of CSLME was investigated on the disruption of F‐actin cytoskeleton in MCF‐7 cells. The actin cytoskeleton is not only provides the physical support and maintenance of the overall cell integrity but also plays a key role in linking the intracellular and extracellular signal transduction and thereby involves in the regulation of cell growth, survival, motility, and death.^44^ Moreover, the actin cytoskeleton is also involved in the process of apoptotic cell death, and series of bioactive molecules/formulations from plant origin such as resveratrol, oleuropein, cucurbitacin E, I & B, 4‐hydroxycoumarin, *Ganoderma lucidum* extracts and so forth are reported to interact with actin and disrupt or alter its normal distribution pattern in different types of cancers.^45^ Results digitized in Figure 6, clearly demonstrate that the CSLME treated MCF‐7 cells had a drastically reduced and disorganized actin cytoskeleton indicating the efficacy of the sample to target multiple sites of apoptosis and cell death.

In concurrence with the anti‐proliferative and apoptosis‐inducing activity of the CSLME, we also investigated the impact of CSLME on cell cycle using flow cytometry study. The outcomes of this cell cycle analysis revealed that CSLME prevents the progression of the cell cycle by significantly limiting the main cell pool in the G2/M phase and there is significant difference in number of cells arrested at G2/M phase in control and CSLME treated cells (Figure 7), indicating that the cell cycle of MCF‐7 is adversely affected by the CSLME.

As a scientific quest and curiosity, and inspired with the impressive results of the CSLME in various assays, the activity of CSLME was also evaluated as a possible inhibitor of angiogenesis using a CAM assay, which essentially mimics the tumor vasculature. Tumor angiogenesis is a feature of cancer and more than 11 anti‐angiogenic drugs approved by the FDA are currently prescribed for the management of the number of cancers.^46^ The result of the CAM assay demonstrates that, CSLME (89.90%) possess the significant antiangiogenic activity and was able to decrease the blood vessel proliferation in chick embryo by decreasing progressively the size, length, number as well as the junctions of tubule complexes in CSLME treated CAMs (Figure 8). The anti‐angiogenic property of CSLME can be attributed to the phytoconstituents present in the CSLME as numerous phytoconstituents like polyphenols, flavonoids, terpenoids, alkaloids, and so forth have been reported to inhibit angiogenesis.^47^

After confirming the apoptosis‐inducing ability of CSLME by performing various assays, the immunoblotting analysis of Bcl2 family proteins such as Bax, Bad (pro‐apoptotic), and Bcl2 (anti‐apoptotic) along with apoptosis‐related enzymes such as caspases (caspase‐9) and PARP‐1 was carried out. Sizable literature has accumulated which describes the regulation of the intrinsic pathway of apoptosis by the Bcl‐2 protein (B‐cell lymphoma‐2) family members, consisting of different pro‐apoptotic and anti‐apoptotic Bcl‐2 proteins.^48^ Bcl‐2 proteins mediate their anti‐apoptotic action by inhibiting pro‐apoptotic players such as Bcl‐2, Bax, and Bak.^49^ Bcl‐2 also positively drives apoptotic cell death by associated cell death agonist (Bad) protein by forming a heterodimer with Bcl‐xL, Bcl‐2, and Bcl‐W, which consequently leads to reversal of their cell death repressor activity.^50^ The immunoblotting result summarized in Figure 9, indicates the significant down regulation of anti‐apoptotic protein Bcl‐2, while considerable up‐regulation of pro‐apoptotic proteins like Bax and Bad were noticed in CSLME treated MCF‐7 cells as opposed to the control set. The outcomes have demonstrated that an array of phytochemicals of CSLME targets the Bcl‐2 family proteins that participate in apoptosis cell death directly.

Caspases are the enzymes belongs to the class of cysteine proteins, (cysteine aspartyl‐specific proteases) and play an important role in apoptosis by cleavage of hundreds of target proteins leading to successful cell death. More specifically, caspases‐2, ‐8, ‐9, and ‐10 acts as an initiator caspase, whereas caspase‐3, ‐6, and ‐7 acts as executioner caspases. Essentially, the executioner caspases cleave the target proteins which ultimately lead to cell death.^48^, ^49^ Among the set of enzymes involved in cellular functioning and survival, poly (ADP‐ribose) polymerase‐1 (PARP‐1) happens to be one of the key enzymes in this sequel. The physiological role of PARP‐1 lies in the repertoire of functions in repairing the damaged DNA by adding poly (ADP ribose) polymers in response to different cellular stresses. While linking the role of PARP‐1 with cell apoptosis, interestingly, PARP‐1 acts as one of several reported cellular substrates of caspases. Of note, successful cleavage of PARP‐1 by caspases has been identified as an early biomarker of successful proapoptotic signaling and apoptotic death of cells.^51^, ^52^ In fact, MCF‐7 originated from ATCC is a caspase‐3 deficient cell line and do not possess a functional caspase‐3. However, there are study reports that describe the apoptosis induced changes in cell morphology and DNA fragmentation in MCF‐7 cells which are independent of caspase‐3. The reports describe that the caspase‐3 independent DNA fragmentation and apoptosis is mediated by effector caspases such as caspase‐6 and 7.^53^ Results obtained demonstrated the cleavage of caspase‐9 and PARP‐1, which focus the possible ability of CSLME phytochemicals toward the activation of intrinsic pathway of apoptosis leading to cell death.

It is well known that inflammation plays a vital role in regulation of various physiological and pathological conditions through diverse molecular pathways.^54^ Accumulating evidences suggest that inflammation also plays crucial role in controlling the initiation and progression of various cancers including breast cancer.^55^ Majority of the inflammatory responses in cancer cells are regulated mainly by two transcription factors such as NF‐κB and STAT3.^56^ A proinflammatory cytokine, tumor necrosis factor (TNF) mediates phosphorylation and activation of NF‐κB which controls cell survival and proliferation of various cancer cells.^57^ However, inhibition of NF‐κB leads to the up‐regulation of apoptotic genes in cancer cells.^57^, ^58^ It was also shown that, NF‐κB pathway plays a crucial role in controlling various aspects of cancer progression such as angiogenesis, metastasis, and therapeutic resistance.^59^ Hence, targeting NF‐κB pathway is one of the promising approaches in effective management of breast cancer. Moreover, a variety of plant derived metabolites have demonstrated inhibitory activity against NF‐κB pathway.^54^ Hence, we hypothesized that CSLME may down regulate the activation of an NF‐κB, which leads to decreased survival and anti‐tumorigenic activity in MCF‐7 cells. Our data suggests that CSLME down regulates both activation and expression of proinflammatory transcription factor such as NF‐κBP65 that may contribute toward anti‐tumorigenic activity in MCF‐7 cells.

Nontoxic or minimum toxicity is the basic prerequisite for testing the drug candidate under in vivo settings. Therefore, to authenticate the safety and nontoxic nature of CSLME, an acute oral toxicity study was conducted on albino Wistar rats. Nontoxic effects of plant extracts are mostly assessed by using acute oral toxicity experiments.^60^ In this study, there was no toxicity and mortality was observed in animals even at a higher dosage of CSLME (2000 mg/kg). Thus, CSLME even at 2000 mg/kg may be considered safe. It is reported that any pharmaceutical drug having an oral LD_50_ higher than 1000 mg/kg can be recognized as safe.^61^

The way that the cytotoxic effects of CSLME have been confirmed initially by in vitro methods; it is of principal necessity to validate their uniformity using in vivo study. Therefore, the anti‐breast cancer efficacy of CSLME was confirmed further by in vivo studies using the MCF‐7 xenografts developed in NOD‐SCID mice. The results of the in vivo study demonstrated that a low dose of CSLME (25 mg/kg body weight) significantly suppressed MCF‐7 tumor growth with little reduction in the weight of tumor‐bearing NOD‐SCID mice (Figure 10). The sensitivity of MCF‐7 tumor xenografts was in agreement with the in vitro cell culture studies which demonstrated inhibition of MCF‐7 cells by CSLME (IC_50_ 20 μg/ml). Although the CSLME is a crude extract, however, owing to the impressive anti‐tumor activity at lower doses under in vivo settings, there is further scope for identification and isolation of novel lead/s for the management of hormone‐dependent breast cancer.

Overall, the various assays and preclinical studies carried out to address the efficacy of CSLME as a potential inhibitor of MCF‐7 breast cancer cells, clearly establish the significance of *C. swietenia* as an important natural resource, which perhaps can be explored further for the identification of novel bioactive anticancer leads. Although indeed it seems difficult to establish the structure–activity relationship with the studied biological parameters owing to the diversity of the metabolites, however, it is in vitro and in vivo anti‐breast cancer activity in MCF‐7 cells could be associated with the involvement of different phytochemicals in CSLME. Previous reports described the anticancer potential of some of the phytochemicals identified in CSLME. The natural coumarin compound for instance is 7‐methoxy‐8‐(3‐methyl‐2‐en‐1‐yl)‐2H‐chromen‐2‐one (Osthole: in CSLME). Osthole has inspired researchers for its widespread pharmacological activities. There is an extensive elucidation of the chemotherapeutic and chemopreventive potential of osthole against various cancer cells such as, leukemia HL‐60, prostatic cancer PC3, cervical cancer Hela, breast cancer MDA‐MB‐231, ovarian cancer SKOV3, epidermal cancer, lung cancer A549 cells, and hepatocellular HepG2 cells.^62^ Other CSLME phytoconstituents like 4‐methoxy‐7H‐furo[3,2‐g]chromen‐7‐one (Bergaptan) has been reported to induce the apoptosis in MCF‐7 cell line.^63^ Another compound like *N*‐hexadecanoic acid (palmitic acid) has been reported to exert antiproliferation effects against human colorectal carcinoma (HCT‐116) cells and human leukemic cell line MOLT‐4.^64^, ^65^

In short, the current results indicated CSLME's potential activities against MCF‐7 breast cancer cells. Data from the preliminary investigation might encourage the research community for undertaking various anticancer mechanisms of CSLME and perhaps facilitate the development of promising and safe anti‐breast cancer therapeutic agents.

## 5 CONCLUSION

The aim of the present investigation was to explore the potential of *C. swietenia* using MCF‐7 cells as a model of hormone dependent breast cancer. In summary, the outcome of the present investigation clearly demonstrated the potential of CSLME as an effective inhibitor of cell growth, proliferation, cell migration, colony formation, and angiogenesis. While unraveling the possible mechanism of action, it was found that CSLME induced apoptosis (most probably via intrinsic pathway) by nuclear fragmentation, cell cycle arrest and targeted cytoskeleton as evidenced form F‐actin derangement. Interestingly, CSLME also down regulated NF‐κB in MCF‐7 cells, which happens to be a key player in controlling various aspects of cancer progression such as angiogenesis, metastasis, and therapeutic resistance. The in vivo acute oral toxicity study conducted as per OECD 423 revealed the nontoxic nature of CSLME. The in vitro cytotoxicity of CSLME was validated under in vivo setting by developing MCF‐7 xenografts in NOD‐SCID mice model. As far as we know; this is the first kind of such study report describing the anti‐breast cancer potential of *C. swietenia* against MCF‐7 cells with the possible mechanism of actions. The study outcome may also inspire further research toward isolation and identification of novel leads from *C. swietenia* against cancer in general and particularly against breast cancer with multiple mechanisms of action as the detailed molecular effect are not fully investigated.

## CONFLICT OF INTEREST

The authors have stated explicitly that there are no conflicts of interest in connection with this article.

## AUTHOR CONTRIBUTIONS

**Sonali Kamble:** Data curation (lead); formal analysis (lead); investigation (lead); methodology (lead); writing – original draft (equal). **Jasoda Choudhari:** Investigation (supporting); methodology (supporting). **Ramakrishna Nimma:** Investigation (supporting); methodology (supporting). **Kumar TVS:** Investigation (supporting); methodology (supporting). **Kapil Patil:** Investigation (supporting). **Shrikant Hese:** Investigation (supporting); methodology (supporting). **Bhaskar Dawane:** Supervision (supporting).

## ETHICS STATEMENT

Animal studies and procedures were approved and monitored by the Institutional Animal Ethics Committee (IAEC) of Pinnacle Biomedical Research Institute (PBRI), Bhopal, India (CPCSEA Reg. No. 1824/PO/ERe/S/15/CPCSEA) and Institutional Review Board (IRB) of the Advanced Centre for Treatment Research and Education in Cancer (ACTREC), Tata Memorial Centre, Kharghar, Navi Mumbai, India. All the institutional guidelines for the care and use of animals were followed.

## Data Availability

The data that support the findings of this study are available on request from the corresponding author.

## References

[1] Zeeneldin AA , Ramadan M , Gaber AA , Taha FM . Clinico‐pathological features of breast carcinoma in elderly Egyptian patients: a comparison with the non‐elderly using population‐based data. J Egypt Natl Canc Inst. 2013;25(1):5‐11. 23499201 10.1016/j.jnci.2012.10.003

[2] Najmuddin SUFS , RomLi MF , Hamid M , Alitheen NB , Rahman NMANA . Anti‐cancer effect of *Annona muricata* Linn leaves crude extract (AMCE) on breast cancer cell line. BMC Complement Altern Med. 2016;16(1):311. 27558166 10.1186/s12906-016-1290-y PMC4997662

[3] Utage BG , Patole MS , Nagvekar PV , Kamble SK , Gacche RN . *Prosopis juliflora* (Sw.), DC induces apoptosis and cell cycle arrest in triple negative breast cancer cells: in vitro and in vivo investigations. Oncotarget. 2018;9(54):30304‐30323. 30100991 10.18632/oncotarget.25717 PMC6084402

[4] Syed FQ , Elkady AI , Mohammed FA , Mirza MB , Hakeem KR , Alkarim S . Chloroform fraction of Foeniculum vulgare induced ROS mediated, mitochondria‐caspase‐dependent apoptotic pathway in MCF‐7, human breast cancer cell line. J Ethnopharmacol. 2018;218:16‐26. 29474902 10.1016/j.jep.2018.02.029

[5] Devi PS , Kumar MS , Das SM . Evaluation of anti‐proliferative activity of red sorghum bran anthocyanin on a human breast cancer cell line (mcf‐7). Int J Breast Cancer. 2011;2011:1‐6. 10.4061/2011/891481 PMC3262581 22312562

[6] Akram M , Iqbal M , Daniyal M , Khan AU . Awareness and current knowledge of breast cancer. Biol Res. 2017;50(1):33. 28969709 10.1186/s40659-017-0140-9 PMC5625777

[7] Graidist P , Martla M , Sukpondma Y . Cytotoxic activity of *Piper cubeba* extract in breast cancer cell lines. Nutrients. 2015;7(4):2707‐2718. 25867951 10.3390/nu7042707 PMC4425168

[8] Newman DJ , Cragg GM . Natural products as sources of new drugs from 1981 to 2014. J Nat Prod. 2016;79(3):629‐661. 26852623 10.1021/acs.jnatprod.5b01055

[9] Goyal S , Gupta N , Chatterjee S , Nimesh S . Natural plant extracts as potential therapeutic agents for the treatment of cancer. Curr Top Med Chem. 2017;17(2):96‐106. 27237328 10.2174/1568026616666160530154407

[10] Ekor M . The growing use of herbal medicines: issues relating to adverse reactions and challenges in monitoring safety. Front Pharmacol. 2014;4:177. 24454289 10.3389/fphar.2013.00177 PMC3887317

[11] Atanasov AG , Waltenberger B , Pferschy‐Wenzig EM , et al. Discovery and resupply of pharmacologically active plant‐derived natural products: a review. Biotechnol Adv. 2015;33(8):1582‐1614. 26281720 10.1016/j.biotechadv.2015.08.001 PMC4748402

[12] Badmus JA , Ekpo OE , Hussein AA , Meyer M , Hiss DC . Anti proliferative and apoptosis induction potential of the methanolic leaf extract of *Holarrhena floribunda* (G. Don). Evid Based Complement Alternat Med. 2015;2015:1‐11. 10.1155/2015/756482 PMC4377504 25861368

[13] Saraydin SU , Tuncer E , Tepe B , et al. Anti tumoral effects of *Melissa officinalis* on breast cancer in vitro and in vivo. Asian Pac J Cancer Prev. 2012;13(6):2765‐2770. 22938456 10.7314/apjcp.2012.13.6.2765

[14] Deb NK , Singh A , Rathore DS , Dash GK , Deb J . Pharmacognostic studies of the stem bark of *Chloroxylon swietenia* DC. Indian J Pharm Biol Res. 2015;3(04):1‐5.

[15] Reddy A . Use of various bio‐fencing plants in the control of human diseases by the Lambada Tribe inhabiting Nalgonda District, Andhra Pradesh, India. Ethnobot Leafl. 2008;2008(1):67.

[16] Harwansh RK , Dangi NSJ . The medicinal plant *Chloroxylon swietenia* as a potential source for agricultural crop protection against aphides (Brown Planthopper), *Nilapurvata lugens*; (Stal) (Hemiptera:Delphacidae) as pesticides. J Pharm Res. 2009;2(5):978‐982.

[17] Melmari S , Jayaraj M . Pharmacognostic and phytochemical investigation on stem bark of chloroxylon swietenia DC. An ethnomedicinally important medicinal tree. Int J Pharm Sci Res. 2015;6(2):825.

[18] Balasubramani G , Ramkumar R , Krishnaveni N , et al. GC‐MS analysis of bioactive components and synthesis of gold nanoparticle using *Chloroxylon swietenia* DC leaf extract and its larvicidal activity. J Photochem Photobiol B. 2015;148:1‐8. 25854160 10.1016/j.jphotobiol.2015.03.016

[19] Deepti K , Umadevi P , Vijayalakshmi G . Antimicrobial activity and phytochemical analysis of *Morinda tinctoria* Roxb. leaf extracts. Asian Pac. J Trop Biomed. 2012;2(3):S1440‐S1442.

[20] Munro B , Vuong QV , Chalmers AC , Goldsmith CD , Bowyer MC , Scarlett CJ . Phytochemical, antioxidant and anti‐cancer properties of *Euphorbia tirucalli* methanolic and aqueous extracts. Antioxidants. 2015;4(4):647‐661. 26783950 10.3390/antiox4040647 PMC4712938

[21] Kamble VT , Sawant AS , Sawant SS , et al. Synthesis and evaluation of new 4‐chloro‐2‐(3‐chloro‐4‐fluorophenyl)‐5‐(aliphatic/cyclic saturated amino) pyridazin‐3 (2H)‐one derivatives as anticancer, antiangiogenic, and antioxidant agents. Arch Pharm. 2015;348(5):338‐346. 10.1002/ardp.201400442 25846009

[22] Kamble S , Utage B , Mogle P , et al. Evaluation of curcumin capped copper nanoparticles as possible inhibitors of human breast cancer cells and angiogenesis: a comparative study with native curcumin. AAPS PharmSciTech. 2016;17(5):1030‐1041. 26729534 10.1208/s12249-015-0435-5

[23] Wu GS , Song YL , Yin ZQ , et al. Ganoderiol A‐enriched extract suppresses migration and adhesion of MDA‐MB‐231 cells by inhibiting FAK‐SRC‐paxillin cascade pathway. PLoS One. 2013;8(10):e76620. 24204647 10.1371/journal.pone.0076620 PMC3812178

[24] He QY , Wang R , Sun XC . Cytotoxicity of methanolic extract of Swertia petiolata against gastric cancer cell line SNU‐5 is via induction of apoptosis. S Afr J Bot. 2017;109:196‐202.

[25] Monga J , Pandit S , Chauhan RS , Chauhan CS , Chauhan SS , Sharma M . Growth inhibition and apoptosis induction by (+)‐Cyanidan‐3‐ol in hepatocellular carcinoma. PLoS One. 2013;8(7):e68710. 23894334 10.1371/journal.pone.0068710 PMC3722203

[26] Majeed R , Reddy MV , Chinthakindi PK , et al. Bakuchiol derivatives as novel and potent cytotoxic agents: a report. Eur J Med Chem. 2012;49:55‐67. 22245048 10.1016/j.ejmech.2011.12.018

[27] Chothiphirat A , Nittayaboon K , Kanokwiroon K , Srisawat T , Navakanitworakul R . Anticancer potential of fruit extracts from *Vatica diospyroides* Symington type SS and their effect on program cell death of cervical cancer cell lines. Sci World J. 2019;2019:1‐9. 10.1155/2019/5491904 PMC6500633 31118873

[28] Pumiputavon K , Chaowasku T , Saenjum C , et al. Cell cycle arrest and apoptosis induction by methanolic leaves extracts of four Annonaceae plants. BMC Complement Altern Med. 2017;17(1):294. 28583139 10.1186/s12906-017-1811-3 PMC5460496

[29] Gacche RN , Shegokar HD , Gond DS , Yang Z , Jadhav AD . Evaluation of selected flavonoids as antiangiogenic, anticancer, and radical scavenging agents: an experimental and in silico analysis. Cell Biochem Biophys. 2011;61(3):651‐663. 21830125 10.1007/s12013-011-9251-z

[30] Han SY , Zhao W , Sun H , et al. Marsdenia tenacissima extract enhances gefitinib efficacy in non‐small cell lung cancer xenografts. Phytomedicine. 2015;22(5):560‐567. 25981922 10.1016/j.phymed.2015.03.001

[31] Mokhtari RB , Baluch N , Tsui MKH , et al. Acetazolamide potentiates the anti‐tumor potential of HDACi, MS‐275, in neuroblastoma. BMC Cancer. 2017;17(1):156. 28235409 10.1186/s12885-017-3126-7 PMC5326494

[32] Elmore S . Apoptosis: a review of programmed cell death. Toxicol Pathol. 2017;35(4):495‐516. 10.1080/01926230701320337 PMC2117903 17562483

[33] Torre LA , Islami F , Siegel RL , Ward EM , Jemal A . Global cancer in women: burden and trends. Cancer Epidemiol Biomarkers Prev. 2017;26(4):444‐457. 28223433 10.1158/1055-9965.EPI-16-0858

[34] Ghasemzadeh A , Ghasemzadeh N . Flavonoids and phenolic acids: role and biochemical activity in plants and human. J Med Plant Res. 2011;5(31):6697‐6703.

[35] Chahar MK , Sharma N , Dobhal MP , Joshi YC . Flavonoids: a versatile source of anticancer drugs. Pharmacogn Rev. 2011;5(9):1‐12. 22096313 10.4103/0973-7847.79093 PMC3210013

[36] Iqbal J , Abbasi BA , Mahmood T , et al. Plant‐derived anticancer agents: a green anticancer approach. Asian Pac J Trop Biomed. 2017;7(12):1129‐1150.

[37] Leyva‐Peralta MA , Robles‐Zepeda RE , Garibay‐Escobar A , Ruiz‐Bustos E , Alvarez‐Berber LP , Gálvez‐Ruiz JC . In vitro anti‐proliferative activity of *Argemone gracilenta* and identification of some active components. BMC Complement Altern Med. 2015;15(1):13. 25652581 10.1186/s12906-015-0532-8 PMC4321710

[38] Rosangkima G , Jagetia GC . In vitro anticancer screening of medicinal plants of Mizoram state, India, against dalton's lymphoma, mcf‐7 and hela cells. Int J Recent Sci Res. 2015;6(8):5648‐5653.

[39] Rafehi H , Orlowski C , Georgiadis GT , Ververis K , El‐Osta A , Karagiannis TC . Clonogenic assay: adherent cells. J Vis Exp. 2011;49:e2573. 10.3791/2573 PMC3197314 21445039

[40] Martin TA , Ye L , Sanders AJ , Lane J , Jiang WG . Cancer invasion and metastasis: molecular and cellular perspective. Madame Curie Bioscience Database. *Landes Bioscience*; 2013.

[41] Kapinova A , Kubatka P , Golubnitschaja O , et al. Dietary phytochemicals in breast cancer research: anticancer effects and potential utility for effective chemoprevention. Environ Health Prev Med. 2018;23(1):1‐18. 30092754 10.1186/s12199-018-0724-1 PMC6085646

[42] Rahman SNSA , Wahab NA , Abd Malek SN . In vitro morphological assessment of apoptosis induced by antiproliferative constituents from the rhizomes of *Curcuma zedoaria* . Evid Based Complement Alternat Med. 2013;2013:1‐14. 10.1155/2013/257108 PMC3671673 23762112

[43] Majid MZ , Mohamad Zaini Z , Abdul RF . Apoptosis‐inducing effect of three medicinal plants on oral cancer cells KB and ORL‐48. Sci World J. 2014;2014:1‐8. 10.1155/2014/125353 PMC4134791 25147833

[44] Burridge K , Wennerberg K . Rho and Rac take center stage. Cell. 2004;116(2):167‐179. 14744429 10.1016/s0092-8674(04)00003-0

[45] Hermenean A , Ardelean A . Targeting the cytoskeleton with plant‐bioactive compounds in cancer therapy. In: Jimenez‐Lopez JC , ed. Cytoskeleton—Structure, Dynamics, Function and Disease. IntechOpen; 2017:315‐332.

[46] Gacche RN , Assaraf YG . Redundant angiogenic signaling and tumor drug resistance. Drug Resist Updat. 2018;36:47‐76. 29499837 10.1016/j.drup.2018.01.002

[47] Jeong SJ , Koh W , Lee EO , et al. Antiangiogenic phytochemicals and medicinal herbs. Phytother Res. 2011;25(1):1‐10. 20564543 10.1002/ptr.3224

[48] Lopez J , Tait SWG . Mitochondrial apoptosis: killing cancer using the enemy within. Br J Cancer. 2015;112(6):957‐962. 25742467 10.1038/bjc.2015.85 PMC4366906

[49] Zaman S , Wang R , Gandhi V . Targeting the apoptosis pathway in hematologic malignancies. Leuk Lymphoma. 2014;55(9):1980‐1992. 24295132 10.3109/10428194.2013.855307 PMC4152229

[50] Kale J , Osterlund EJ , Andrews DW . BCL‐2 family proteins: changing partners in the dance towards death. Cell Death Differ. 2018;25(1):65‐80. 29149100 10.1038/cdd.2017.186 PMC5729540

[51] Kaufmann SH , Desnoyers S , Ottaviano Y , Davidson NE , Poirier GG . Specific proteolytic cleavage of poly(ADP‐ribose) polymerase: an early marker of chemotherapy‐induced apoptosis. Cancer Res. 1993;53(17):3976‐3985. 8358726

[52] Chaitanya GV , Alexander JS , Babu PP . PARP‐1 cleavage fragments: signatures of cell‐death proteases in neurodegeneration. J Cell Commun Signal. 2010;8(1):1‐11. 10.1186/1478-811X-8-31 PMC3022541 21176168

[53] McGee MM , Hyland E , Campiani G , Ramunno A , Nacci V , Zisterer DM . Caspase‐3 is not essential for DNA fragmentation in MCF‐7 cells during apoptosis induced by the pyrrolo‐1,5‐benzoxazepine, PBOX‐6. FEBS Lett. 2002;515(1–3):66‐70. 11943196 10.1016/s0014-5793(02)02440-7

[54] Aggarwal BB , Prasad S , Reuter S , et al. Identification of novel anti‐inflammatory agents from Ayurvedic medicine for prevention of chronic diseases: “reverse pharmacology” and "bedside to bench" approach. Curr Drug Targets. 2011;12(11):1595‐1653. 21561421 10.2174/138945011798109464 PMC3170500

[55] Singh N , Baby D , Rajguru JP , Patil PB , Thakkannavar SS , Pujari VB . Inflammation and cancer. Ann Afr Med. 2019;18(3):121‐126. 31417011 10.4103/aam.aam_56_18 PMC6704802

[56] Li F , Shanmugam MK , Chen L , et al. Garcinol, a polyisoprenylated benzophenone modulates multiple proinflammatory signaling cascades leading to the suppression of growth and survival of head and neck carcinoma. Cancer Prev Res (Phila). 2013;6(8):843‐854. 23803415 10.1158/1940-6207.CAPR-13-0070

[57] Grewal IS . Therapeutic Targets of the TNF Superfamily. Advances in Experimental Medicine and Biology. Springer; 2009:1‐7. 10.1007/978-0-387-89520-8_1 19760063

[58] Takada Y , Ichikawa H , Pataer A , Swisher S , Aggarwal BB . Genetic deletion of PKR abrogates TNF‐induced activation of I κ B α kinase, JNK, Akt and cell proliferation but potentiates p44/p42 MAPK and p38 MAPK activation. Oncogene. 2007;26(8):1201‐1212. 16924232 10.1038/sj.onc.1209906

[59] Xia Y , Shen S , Verma IM . NF‐κB, an active player in human cancers. Cancer Immunol Res. 2014;2(9):823‐830. 25187272 10.1158/2326-6066.CIR-14-0112 PMC4155602

[60] Subha D , Geetha N . Evaluation of acute toxicity of the methanolic extract of *Tanacetum parthenium* L. in albino wistar rats. J Sci Innov Res. 2017;6:113‐115.

[61] Kifayatullah M , Mustafa MS , Sengupta P , Sarker MMR , Das A , Das SK . Evaluation of the acute and sub‐acute toxicity of the ethanolic extract of *Pericampylus glaucus* (Lam.) Merr. in BALB/c mice. J Acute Dis. 2015;4(4):309‐315.

[62] Zhang ZR , Leung WN , Cheung HY , Chan CW . Osthole: a review on its bioactivities, pharmacological properties, and potential as alternative medicine. Evid Based Complement Alternat Med. 2015;2015:1‐10. 10.1155/2015/919616 PMC4515521 26246843

[63] Tomasz Kubrak T , RafałPodgórski R , Monika SM . Natural and synthetic coumarins and their pharmacological activity. Eur J Clin Exp Med. 2017;15(2):169‐175.

[64] Ravi L , Krishnan K . Research article cytotoxic potential of N‐hexadecanoic acid extracted from Kigelia pinnata leaves. Asian J Cell Biol. 2017;12(1):20‐27.

[65] Harada H , Yamashita U , Kurihara H , Fukushi E , Kawabata J , Kamei Y . Antitumor activity of palmitic acid found as a selective cytotoxic substance in a marine red alga. Anticancer Res. 2002;22(5):2587‐2590. 12529968

